# A new species of *Enteromius* (Actinopterygii, Cyprinidae, Smiliogastrinae) from the Awash River, Ethiopia, and the re-establishment of *E. akakianus*

**DOI:** 10.3897/zookeys.902.39606

**Published:** 2020-01-13

**Authors:** Gernot K. Englmaier, Genanaw Tesfaye, Nina G. Bogutskaya

**Affiliations:** 1 University of Graz, Institute of Biology, Universitätsplatz 2, A-8010 Graz, Austria University of Graz Graz Austria; 2 National Fisheries and Aquatic Life Research Centre, P.O.Box: 64, Sebeta, Ethiopia National Fisheries and Aquatic Life Research Centre Sebeta Ethiopia; 3 Natural History Museum Vienna, Burgring 7, A-1010 Vienna, Austria Natural History Museum Vienna Vienna Austria

**Keywords:** East Africa, Main Ethiopian Rift, morphology, CO1 and cytb sequences, zoogeography

## Abstract

In the present study, populations of small-sized smiliogastrin barbs with a thickened and serrated last simple dorsal-fin ray distributed in the Main Ethiopian Rift were analysed. An integrated approach combining genetic markers and a variety of morphological methods based on a wide set of characters, including osteology and sensory canals, proved to be very productive for taxonomy in this group of fishes. The results showed that Ethiopian *Enteromius* species with a serrated dorsal-fin ray are distant from the true *E. paludinosus* (with *E. longicauda* as a synonym) and the so-called *E. paludinosus* complex involves several supposedly valid species with two distinct species occurring in the Main Ethiopian Rift area. A new species, *Enteromius yardiensis***sp. nov.**, is described from the Afar Depression in the north-eastern part of the Northern Main Ethiopian Rift. *Enteromius akakianus* is resurrected as a valid species including populations from the Central Main Ethiopian Rift (basins of lakes Langano, Ziway, and Awasa). No genetic data were available for *E. akakianus* from its type locality. *Enteromius yardiensis***sp. nov.** is clearly distant from *E. akakianus* from the Central Main Ethiopian Rift by CO1 and cytb barcodes: pairwise distances between the new species and the Ethiopian congeners were 5.4 % to 11.0 %. Morphologically, the new species most clearly differs from all examined Ethiopian congeners by three specialisations which are unique in the group: the absence of the anterior barbel, the absence of the medial branch of the supraorbital sensory canal, and few, 1–3, commonly two, scale rows between the lateral line and the anus.

## Introduction

Small-sized smiliogastrin barbs are typical representatives of the fish fauna in sub-Saharan Africa (Lévêque and Daget 1984; Skelton 2001; Stiassny et al. 2007) with several hundred species distributed in almost all drainages of the continent (Lévêque and Daget 1984). Despite their widespread occurrence, phylogenetic relationships within the group and taxonomy of most species have not been fully resolved yet (Yang et al. 2015; Ren and Mayden 2016; Hayes and Armbruster 2017). In previous taxonomic studies, small-sized African smiliogastrin barbs were commonly referred to as a polyphyletic assemblage named *Barbus* sensu lato (e.g., Greenwood 1962; Berrebi et al. 1996; Golubtsov and Berendzen 2005; Mina et al. 2017). Recent molecular phylogenetic studies of barbeled cypriniforms supported a different opinion (e.g., Karaman 1971; Roberts 2010) that the assemblage of Palearctic true barbs – *Barbus* Daudin, 1805, typified by the species *Cyprinus barbus* Linnaeus, 1758 and close genera – do not occur in sub-Saharan Africa (Tsigenopoulos and Berrebi 2000; Wang et al. 2013; Yang et al. 2015; Ren and Mayden 2016). Though the phylogenetic resolution was considered by some authors as rather limited (Schmidt and Bart 2015; Stiassny and Sakharova 2016), all available genetic data clearly indicate that diploid African taxa belong to a phylogenetically distinct clade, the tribe Smiliogastrini or the subfamily Smiliogastrinae of the family Cyprinidae (in case the subfamily Cyprininae is given the family rank) and are not closely related to *Barbus* sensu stricto (Yang et al. 2015; Ren and Mayden 2016).

Accordingly, the oldest available name for this group, *Enteromius* Cope, 1867, was resurrected at the generic level (Yang et al. 2015). The decision was criticised (Schmidt and Bart 2015; Stiassny and Sakharova 2016; Stiassny et al. 2016; Schmidt et al. 2017) but has been accepted (Schmidt et al. 2018; Mamonekene et al. 2018; Mipounga et al. 2019) based on a summarising review by Hayes and Armbruster (2017) as the first step for taxonomic delimitation of small-sized African smiliogastrin barbs. The phylogenetic trees in Yang et al. (2015) and Ren and Mayden (2016) adopted by Hayes and Armbruster (2017) indicate heterogeneity of the genus, which contains at least two putative distinct genera, Clade *Enteromius* I and Clade *Enteromius* II of Hayes and Armbruster (2017). Which one of the two represents the genus *Enteromius* is not clear because the placement of the type species of *Enteromius* (*E. potamogalis* Cope, 1867) within *Enteromius* sensu lato is still uncertain (Ren and Mayden 2016; Hayes and Armbruster 2017). If the opinion of Roberts (2010) that *E. potamogalis* is closely related to Central and West African taxa is proved to be correct, then the clade *Enteromius* I represents the true *Enteromius.* This issue is beyond the goal of our study and we do not discuss a valid taxonomic name (or the absence of it) for the clade *Enteromius* II of Hayes and Armbruster (2017). We use the genus name *Enteromius* as a convenient taxonomic compromise at the present level of knowledge on phylogenetic interrelationships in the group.

Based on morphology, a supposedly non-monophyletic but readily diagnosable group of *Enteromius* occurs in Ethiopia – comparatively small-sized smiliogastrin barbs with a thickened, segmented only at the tip, and serrated last unbranched ray in the dorsal fin. It includes taxa of the species level originally described under five available names as follows: *E. paludinosus* (Peters, 1852), *E. kerstenii* (Peters, 1868), *E. pleurogramma* (Boulenger, 1902), *E. amphigramma* (Boulenger, 1903) (in Boulenger 1903a) and *E. akakianus* (Boulenger, 1911). They were considered valid species or synonymised in various ways by different authors (Greenwood 1962; Golubtsov and Krysanov 1993; Admassu and Dadebo 1997; Golubtsov et al. 2002; Golubtsov and Krysanov 2003; Golubtsov and Berendzen 2005; De Graaf et al. 2007; Vijverberg et al. 2012; Mina et al. 2017) and commonly divided into two phenotypic groups. One contains *E. paludinosus*-like taxa characterised by the absence of an orange or yellow blotch on the operculum and over 30 total lateral-line scales (*E. pleurogramma*, *E. akakianus* and *E. amphigramma*) and the other, the *E. kerstenii* complex, with species possessing an orange or yellow blotch on the operculum and fewer than 30 total lateral-line scales (Greenwood 1962; Golubtsov and Krysanov 1993; Golubtsov et al. 2002; Golubtsov and Berendzen 2005; Mina et al. 2017).

Morphological observations indicated that most Ethiopian populations of the first group are similar to *E. paludinosus* (Golubtsov and Berendzen 2005). Initially described from the Lower Zambezi River (Peters 1852), *E. paludinosus* was thought to be widely distributed, from South Africa in the south to Ethiopia in the north (Lévêque and Daget 1984; Seegers 1996; Skelton 2001; Marshall 2011). In contrast, genetic studies (De Graaf et al. 2007; Mwita 2013; Schmidt et al. 2017) highlighted distinct differences between groups of populations of E. cf. paludinosus in East Africa. Based on data of Schmidt et al. (2017), Mina et al. (2017) recently re-assigned all Ethiopian populations, commonly identified as *E. paludinosus*, to "*E. pleurogramma* complex" but did not provide any data that could support this conclusion.

Twelve nominal species are synonymised (Seegers 1996; Seegers et al. 2003; Hayes and Armbruster 2017) with *E. paludinosus* originally described from Quellimane, Mozambique (Zambezi River delta). They are as follows (drainage of their type localities in parentheses), all described originally as *Barbus*:

*akakianus* (Akaki River, Awash (endorheic), Ethiopia),

*amphigramma* (Nairobi River, Athi, Kenya); in the original description the location is given as "Nairobi River, Kilimanjaro",

*helleri* Hubbs, 1918 (Athi River, Athi, Kenya),

*ivongoensis* Fowler, 1934 (Ivongo River, Ivongo, South Africa),

*longicauda* Boulenger, 1905 (Zambezi River, Zambezi, Mozambique); replacement name for *B. gibbosus* Peters, 1852, *longicauda* is a noun in apposition, not be changed to agree in gender with the masculine generic name according to Art. 34.2.1. of the International Code of Zoological Nomenclature (International Commission on Zoological Nomenclature 1999),

*macropristis* Boulenger, 1904 (Lake Victoria [Victoria Nyanza], Nile, Kenya),

*macropristis meruensis* Lönnberg, 1907 (River Ngare na nyuki, Nile, Tanzania),

*taitensis* Günther, 1894 (unknown drainage, Taita, Kenya),

*thikensis* Boulenger, 1905 (Thika River, Tana, Kenya),

*tsotsorogensis* Fowler, 1935 (Tsotsoroga Pan, northeastern edge of the Mababe Flats (possibly endorheic), Okawango, Botswana),

*vinciguerraii* Pfeffer, 1896 (Wembere River, Lake Kitangiri basin (endorheic), Tanzania),

*welwitschii* Günther, 1868 (unknown drainage, Huilla and Benguella provinces, Angola).

During recent field trips, samples of small smiliogastrin barbs with a thickened and serrated last unbranched ray in the dorsal fin were collected in central Ethiopia. Preliminary observations showed strong phenotypic variations and suggested an undescribed species of *Enteromius* in the Lower Awash River. In the present paper, we only discuss *E. paludinosus*-like fishes with a serrated dorsal-fin ray. Herein, we present genetic and morphological analyses of Ethiopian samples from the Main Ethiopian Rift endorheic drainages and compare them with type series of *E. akakianus, E. longicauda, E. paludinosus* and *E. pleurogramma* in order to evaluate their taxonomic status and identity.

## Materials and methods

### Sampling and preservation

**Figure 1.**
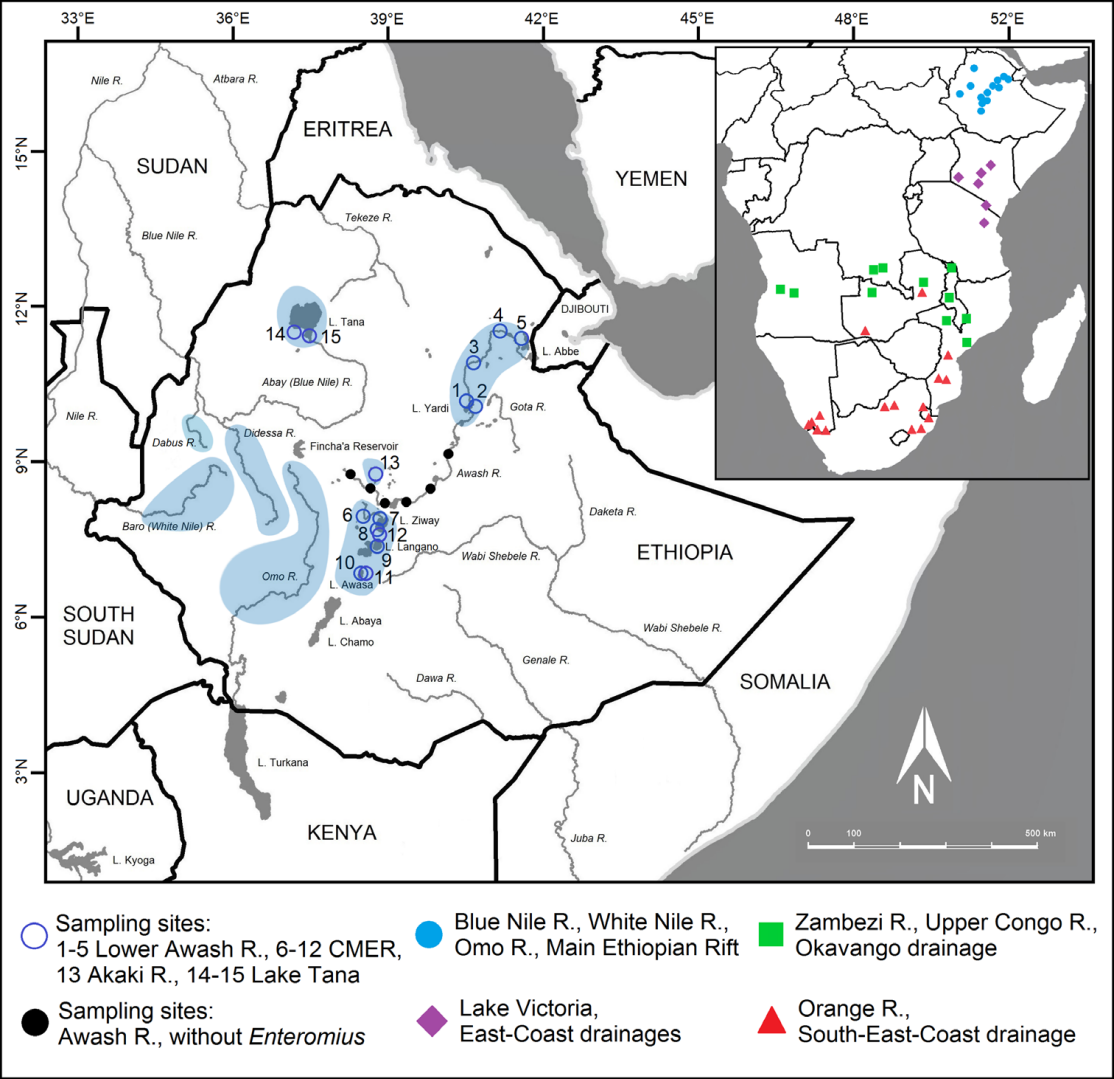
Map of Ethiopia, presenting sampling sites and examined material. Sampling sites: **1–13** Main Ethiopian Rift (**1–5** Lower Awash R., distribution of *Enteromius yardiensis* sp. nov.; **6–12** lakes and rivers in Central Main Ethiopian Rift; **13** Akaki R., type locality of *E. akakianus*); **14–15** Blue Nile drainage, type locality of *E. pleurogramma*. In blue, known populations of small-sized *E. paludinosus*-like smiliogastrin barbs in Ethiopia. Locations in southern Africa included in CO1 and cytb analyses in red, green, and purple. Distribution data for Ethiopian populations from Golubtsov and Berendzen (2005).

During recent field trips (2017–2019), the fish fauna of the Awash River was investigated from the source region in the Chilimo forest to the lakes of the Afar Depression (Englmaier 2018). Sampled localities are given in Fig. 1 including those where no *Enteromius* were found. *Enteromius* were collected (Fig. 1: sample sites 1–10) in endorheic drainages of the Main Ethiopian Rift (MER; same as the Ethiopian Rift Valley of Paugy (2010)) and the Lower Awash River (the Afar Depression). The abbreviation CMER refers to the Central Main Ethiopian Rift (definition and abbreviation follow Bonini et al. (2005)). Length of sampled aquatic segments was between 5 and 20 m (maximum water depth 1.5 m). Collections were made from the main river channel, side arms and shoreline habitats using beach seines (mesh size 1.5 mm) and frame nets (mesh size 1.5 mm). Fish specimens were first euthanised with etheric clove oil (*Eugenia caryophyllata*) diluted in water, and later fixed in 6 % pH neutral formalin or 96 % ethanol. Formalin-preserved specimens were later transferred to ethanol.

Museum samples included specimens deposited in the collections of the Natural History Museum Vienna (**NMW**; Fig. 1: sample sites 1–10); the British Museum of Natural History (**BMNH**; Fig. 1: sample sites 11–15); Museum für Naturkunde, Leibniz-Institut für Evolutions- und Biodiversitätsforschung, Berlin, Germany (**ZMB**); and the fish collection of the National Fisheries and Aquatic Life Research Centre, Sebeta, Ethiopia (**NFALRC**) (see comparative material in Table 1). Data for the type series of the new species are in the species description part. In case of uncertain identification, the term "cf." is used to indicate that a taxon is considered as close or to be compared with another one (Lucas 1986).

**Table 1.** Comparative material. CMER referring to Central Main Ethiopian Rift as defined in text.

Taxon name	Museum number	n	Types	SL, mm	Information
*Barbus akakianus*	BMNH 1908.1.20.85	1	holotype	66.0	Akaki River, Hawash [Awash] system (site 13), Ethiopia, coll. P. Zaphiro.
*Barbus akakianus*	BMNH 1908.1.20.82–283	2	non-types	67.8–88.5	Akaki River, Hawash [Awash] system (site 13), Ethiopia, coll. P. Zaphiro.
*Barbus akakianus*	BMNH 1908.1.20.84	1	non-type	88.1	Dry skeleton, Akaki River, Hawash [Awash] system (site 13), Ethiopia, coll. P. Zaphiro.
*Barbus amphigramma*	BMNH 1902.11.8.24–26	3	syntypes	31.1–34.9	Nairobi River, Kilimanjaro, Tanzania.
*Barbus longicauda*	ZMB 4735	1	lectotype	66.3	Tette [Tete], Revugo, Mozambique
*Barbus longicauda*	ZMB 32377	2	paralectotypes	61.9–67.8	Tette [Tete], Revugo, Mozambique
*Barbus macropristis*	BMNH 1904.5.19.22–23	2	syntypes	98.3–111.6	Lake Victoria, coll. W. Doggett
*Barbus macropristis meruensis*	ZMB 16580	1	syntype	62.1	Meru Nied.: Floden Ngare na nyuki, mount Meru, Tanzania
*Barbus paludinosus*	ZMB 4732	1	lectotype	71.3	Quellimane, Mozambique
*Barbus paludinosus*	ZMB 32375	3	paralectotypes	67.3–70.4	Quellimane, Mozambique
*Barbus paludinosus*	ZMB 4733	4	paralectotypes	42.1–54.7	Quellimane, Mozambique
*Barbus paludinosus*	ZMB 4734	5	paralectotypes	35.9–48.3	Quellimane, Mozambique
*Barbus paludinosus*	ZMB 4738	2	paralectotypes	43.5–46.6	Quellimane, Mozambique
*Barbus paludinosus*	NMW 54476	2	paralectotypes	50.7–65.5	Quellimane, Mozambique
*Barbus paludinosus*	BMNH 1861.3.10.6–7	3	paralectotypes	50.0–53.0	Quellimane, Mozambique, don. Peters
*Barbus pleurogramma*	BMNH 1902.12.13.356	1	syntype	28.5	Unfras River, Lake Tsana [Tana] (site 14), Ethiopia, coll. E. Degen.
*Barbus pleurogramma*	BMNH 1902.12.13.353–355	3	syntypes	30.3–35.4	Bahardar, Lake Tsana [Tana] (site 15), Ethiopia, coll. E. Degen
*Barbus vinciguerraii*	ZMB 14496	10	syntypes	26.3–39.5	Wembere brook, Njagaua, Tanzania
*Enteromius* sp. CMER	BMNH 1903.11.16.10–12	2	non-types	48.1–61.2	Suksuk [Bulbula] R., tributary to Lake Abijata [Abiyata] (site 12), Lake Ziway basin, Ethiopia, coll. C. Erlanger et al.
*Enteromius* sp. CMER	BMNH 1985.7.16.101–105	5	non-types	38.9–55.7	Lake Awasa [Awassa] (site 11), Rift Valley, Ethiopia, don. A. Harrison.
*Enteromius* sp. CMER	NMW 99236	5	non-types	33.8–38.20	Western shore of Lake Ziway (site 7; 8°1’44”N, 38°44’32”E), Ethiopia, 22.05.2018, coll. G.K. Englmaier and G. Tesfaye (and two specimens, both vouchers for CO1 and cytb; MN747020, MN747030, 45.2 mm SL; and MN747021, MN747031, 37.7 mm SL)
*Enteromius* sp. CMER	NMW 99237	2	non-types	38.9–41.0	South-western shore of Lake Ziway (site 8; 7°56’7”N, 38°43’41”E), Ethiopia, 22.05.2018, coll. G.K. Englmaier and G. Tesfaye (and one specimen, C&S, 34.8 mm SL)
*Enteromius* sp. CMER	NMW 99238	4	non-types	42.2–45.4	Western shore of Lake Ziway (site 7; 8°1’44”N, 38°44’32”E), Ethiopia, 22.05.2018, coll. G.K. Englmaier and G. Tesfaye (and one specimen C&S, 39.4 mm SL)
*Enteromius* sp. CMER	NMW 99239	4	non-types	50.4–70.4	Labo River, a tributary of the Meki River (site 6; 8°14’18”N, 38°28’58”E), Lake Ziway basin, Ethiopia, 13.09.2008, coll. F. Wicker and K. Borkenhagen (and one specimen, C&S, 53.4 mm SL)
*Enteromius* sp. CMER	NMW 99260	6	non-types	33.2–46.4	Western shore of Lake Ziway (site 7; 8°1’44”N, 38°44’32”E), Ethiopia, 22.05.2018, coll. G.K. Englmaier and G. Tesfaye
*Enteromius* sp. CMER	NMW 99261	3	non-types	31.0–35.3	South-western shore of Lake Ziway (site 8; 7°56’7”N, 38°43’41”E), Ethiopia, 22.05.2018, coll. G.K. Englmaier and G. Tesfaye
*Enteromius* sp. CMER	NMW 99643	2	non-types	42.4–48.7	Shoreline of Lake Ziway (site 8), Ethiopia, 2018, coll. G. Tesfaye.
*Enteromius* sp. CMER	NMW 99644	10	non-types	43.1–63.1	Lake Langano (site 9), Ethiopia, coll. G. Tesfaye.

### Genetic analyses

Total genomic DNA was extracted from ethanol preserved tissue (fin clips) using the GenElute Mammalian Genomic DNA Miniprep Kit (Sigma-Aldrich, St. Louis, USA). Two mitochondrial regions, cytochrome *c* oxidase subunit 1 (CO1) and cytochrome *b* (cytb), were used for species differentiation. Partial CO1 (approximately 660 bp) was amplified with primers Fish-Co1-F and Fish-Co1-R (Baldwin et al. 2009). Volumetric composition of the PCR mix and thermal setting during 35 cycles of PCR followed Meulenbroek et al. (2018). The partial cytb gene (approximately 650 bp) was amplified with primers L15267 and H15891 (Briolay et al. 1998). The temperature profile was set at 95 °C for 2 min (initial denaturation), followed by 32 cycles of 95 °C for 40 s, 55 °C for 40 s, 72 °C for 1:20 min, and a final extension at 72 °C for 10 min. Gel electrophoresis was used to verify the size of the amplified DNA fragments. PCR products were then sent to Eurofins Genomics (Ebersberg, Germany) for sequencing (Sanger sequencing). Data for specimens used in the genetic analyses and GenBank accession numbers of sequences are in Suppl. material 1, Table S1. We used two different sets of specimens. The first contained data for CO1 (611 bp; 40 specimens) and the second for cytb (520 bp; 66 specimens). Only four specimens were used for both analyses.

CO1 and partial cytb were edited in MEGA7 (Kumar et al. 2016) and aligned with ClustalW. Maximum Likelihood analysis (ML) (1000 bootstrap replications), using RAxML-HPC2 (Stamatakis 2014), and Bayesian Inference analysis (BI), using MrBayes v. 3.2.6 (Ronquist et al. 2012), were performed via the CIPRES Science Gateway (Miller et al. 2011). Both analyses were run under the most general model (GTR+G+I), following the approach of Sayyadzadeh et al. (2015). Two independent runs were performed during BI and Markov chains were run for 5 million generations. Trees were sampled every 100 generations. Tracer v. 1.7.1 (Rambaut et al. 2018) was used to discard the first 25 % as burn-in. The variation between groups (p-distances) was calculated in MEGA7 (Kumar et al. 2016). Asian smiliogastrins were included as outgroups (Yang et al. 2015, Schmidt et al. 2017). FigTree v. 1.4.4 (Rambaut 2018) and CorelDRAW Graphics Suite X7 were used for visualisation.

### Morphological analyses

Data for all examined specimens (total 146) are presented below in the text of the new species description. Comparative material included type specimens (either syntypes or lectotypes and paratypes) of eight nominal species involved in taxonomy of the Ethiopian *Enteromius*.

Therefore, we examined a wide set of morphological characters (Suppl. material 1, Table S2), including shape and number of the axial skeleton elements and infraorbital bones as suggested by Mamonekene et al. (2018).

In total, 31 measurements were made point to point using a digital calliper to the nearest 0.1 mm. The fin insertion is the posterior-most point where the last fin ray connects with the body. Most measurements follow Hubbs and Lagler (1958) and Holčík et al. (1989). Standard length (SL) is measured from the anteriormost point of the head to the posterior margin of the hypurals at midline. Head length (HL) excludes the skin fold on the operculum. Body depth was measured at pelvic-fin origin and maximum caudal-peduncle depth at the anal-fin insertion. Additional measurements of the cranium, jaws and operculum were made point to point from the anteriormost extremity to the posteriormost extremity (lengths), from the uppermost extremity to the lowermost extremity (depths), and between the lateralmost extremities (widths). Length of the cranial roof was measured from the anterior margin of the supraethmoid to the base of the supraoccipital crest. Length of the pelvic splint was also measured (between posterior and anterior extremities). Three measurement of the last unbranched dorsal-fin ray (lengths of lower non-serrated part, serrated part and unsegmented part) were taken from radiographs. Total length of the last unbranched dorsal-fin ray was taken in relatively few specimens because the uppermost segmented part of the ray is often broken.

For morphometric analyses, we used 57 characters, including proportional measurements as specified in Tables 2–3 and Suppl. material 1, Table S3.

Definitions of the used meristic characters, 28, are given in Table 4 and in Suppl. material 1, Table S2. The posterior two branched rays in the dorsal and anal fins were counted as two. As scales are often lost while sampling and preservation, we calculated total number of scales in the lateral series (bearing the lateral-line canal or scale pockets in case of scale loss) including scales at the caudal-fin base and the number of lateral-line scales to the posterior margin of the hypurals at midline. Vertebral counts and terminology follow Naseka (1996) and were taken from radiographs. Terminology of barbels and coding for barbel length follow Lévêque et al. (1987). The sample from Lake Awasa lacks data on five scale counts (Suppl. material 1, Table S3).

Infraorbital bones (io1-io5) and the cephalic sensory canals were examined from alizarin Red S stained (C&S) specimens. The cephalic sensory canal terminology is based on Reno (1969) following Skelton (1980: fig. 3.48) in its application to barbin cyprinids.

Multivariate data analyses included *forward stepwise* discriminant function analysis (DFA), principal component analysis (PCA), cluster analysis (CA) (using the *complete linkage method* with *Euclidean distance*), and multidimensional scaling (MDS). The statistical analyses were performed using Microsoft Excel, Statistica 6.0 (Statistic for Windows. StatSoft) and PAST v. 3.16 (Hammer 1998–2012) software.

In some specimens, due to damage or poor preservation condition, individual measurements could not be obtained; to remain important specimens in the analyses, group means were used to substitute missing data. These cases are highlighted in Suppl. material 1, Table S3.

## Results

### Genetic analyses

**Figure 2.**
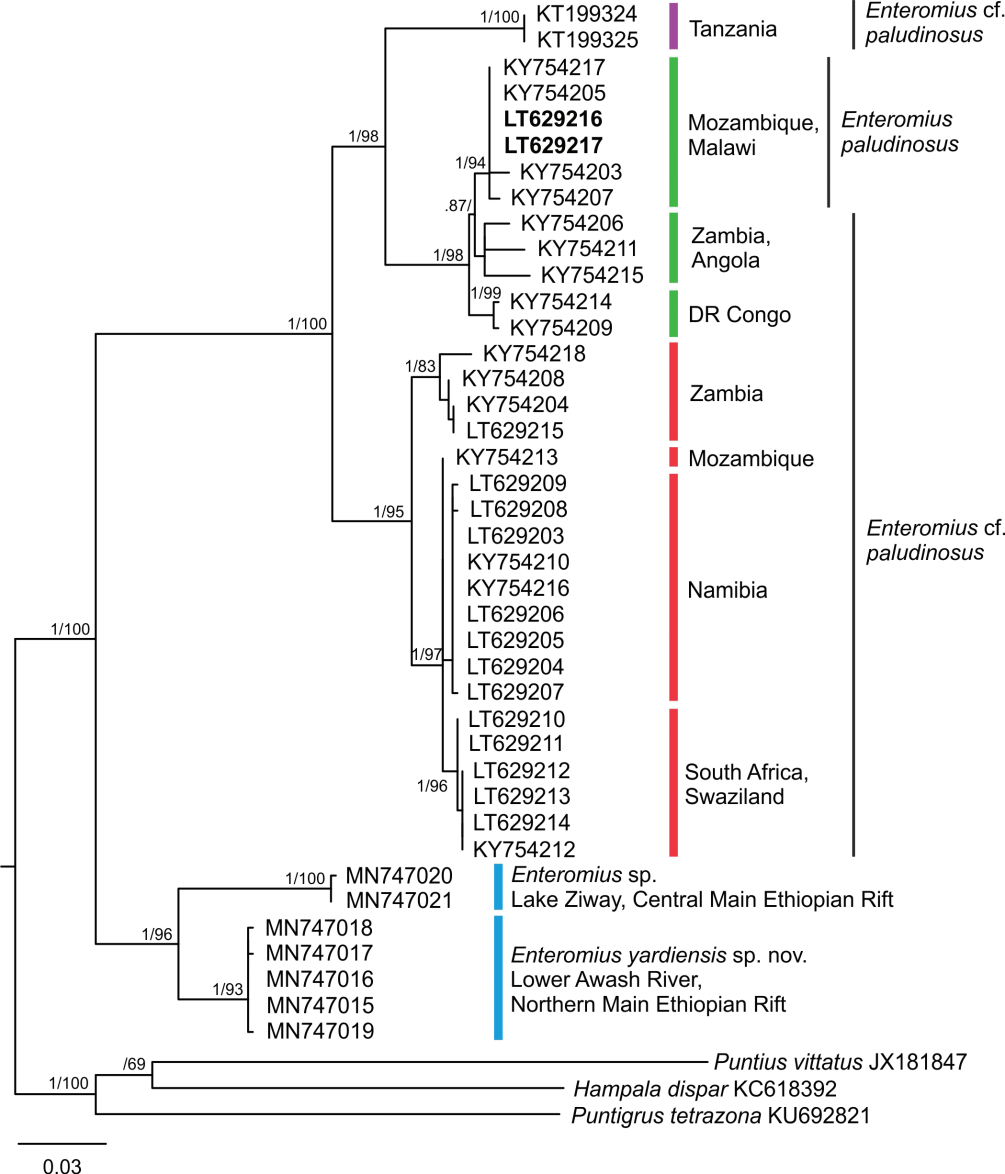
Maximum Likelihood (ML) analysis, 611 bp of CO1. Posterior probabilities from BI analysis and bootstrap (bs) values for ML (1000 bootstrap replications) above and below slash. Values below 0.70/50 considered as collapsed. Colours corresponding to those in Fig. 1. In bold, samples LT629216 and LT629217 representing locality geographically closest to type locality of *Enteromius paludinosus*.

An analysis of 611 bp of the mitochondrial CO1 (Fig. 2) included Ethiopian samples (Lower Awash and Lake Ziway) and *Enteromius* (*E. paludinosus*, E. cf. paludinosus in Fig. 2) from multiple drainage systems in southern Africa. Two specimens from the Lower Zambezi River in Mozambique (LT629216 and LT629217, Tete, type locality of *E. longicauda*) represent a locality geographically closest to the type locality of *E. paludinosus* (Quellimane). The two sister lineages from Ethiopia clustered clearly outside this group (Bayesian posterior probability, BPP 1.00; bootstrap value, bs 96) and the pairwise Euclidean distance between them (the Ziway and the Lower Awash) is 5.4–6.0 %. Pairwise Euclidean distance between Ethiopian and southern African lineages ranged from 10.5 % to 16.3 %. The sample from the Lower Awash is diverged from *E. paludinosus* (Lower Zambezi River) by a mean p-Euclidean distance of 12.0 %.

**Figure 3.**
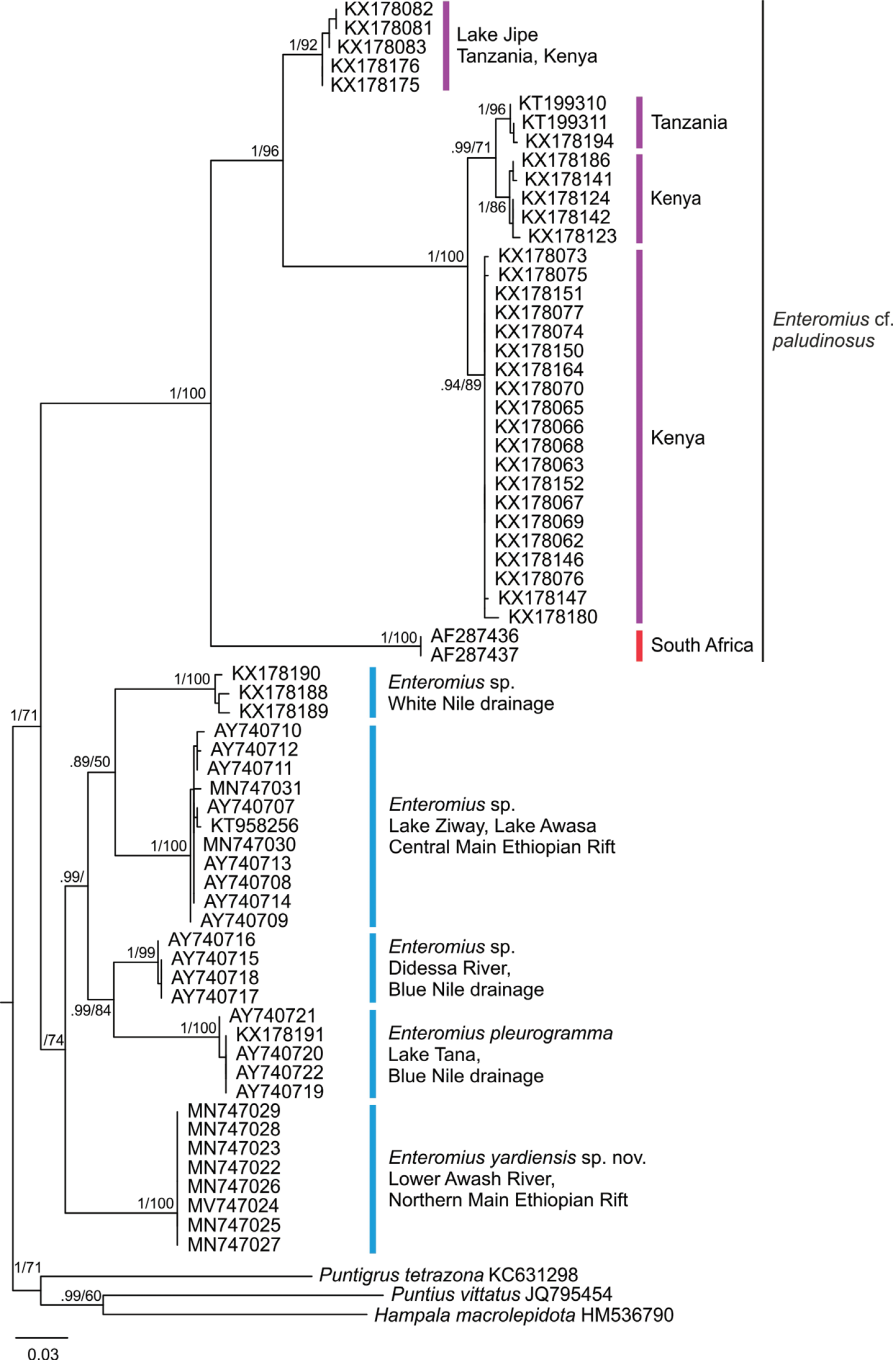
Maximum Likelihood (ML) analysis, 520 bp of partial cytb. Posterior probabilities from BI analysis and bootstrap (bs) values for ML (1000 bootstrap replications) above and below slash. Values below 0.70/50 considered as collapsed. Colours corresponding to those in Fig. 1.

The analysis of 520 bp of the partial cytb included voucher specimens of *E. paludinosus*-like fishes from South Africa, Tanzania, Kenya and Ethiopia (Fig. 3). Considerable genetic divergence was observed within the group of Ethiopian small-sized smiliogastrin barbs with a thickened and serrated dorsal-fin ray. All Ethiopian lineages from the White Nile system, the Blue Nile system and the MER belong to a weakly-supported clade (bs 74) but are clearly separated from specimens in East and South Africa (BPP 1.00, bs 100). Pairwise Euclidean distance between these clades ranged from 12.4 % to 17.8 %. The ML analysis supports a monophyletic Ethiopian clade (Fig. 3) but lacks nodal support from the BI analysis, which revealed an unresolved trichotomy (Suppl. material 2, Fig. S1).

The Lower Awash River sample is a distinct lineage (BPP 1.00, bs 100). Pairwise Euclidean distance (Suppl. material 1, Table S4) between the newly described population from the Lower Awash River and Ethiopian congeners ranged from 8.4 % to 11.0 %, with the lowest divergence to specimens from the CMER and the highest difference to *E. pleurogramma* from Lake Tana. Specimens from Lake Ziway and Lake Awasa do not form differentiated lineages. Pairwise Euclidean distance within this group ranged from 0.0 % to 1.0 %. The highest divergence (1.0 %) is between Lake Awasa (AY740710) and Lake Ziway (MN747031). The two lakes share the same haplotype (AY740708, AY740713, AY740714). The CMER clade is well differentiated from *E. pleurogramma* (9.4 %–10.2 %) and is the closest sister-clade to the White Nile *Enteromius* sp. (8.0 %–9.0 %).

### Morphological analyses

**Figure 4.**
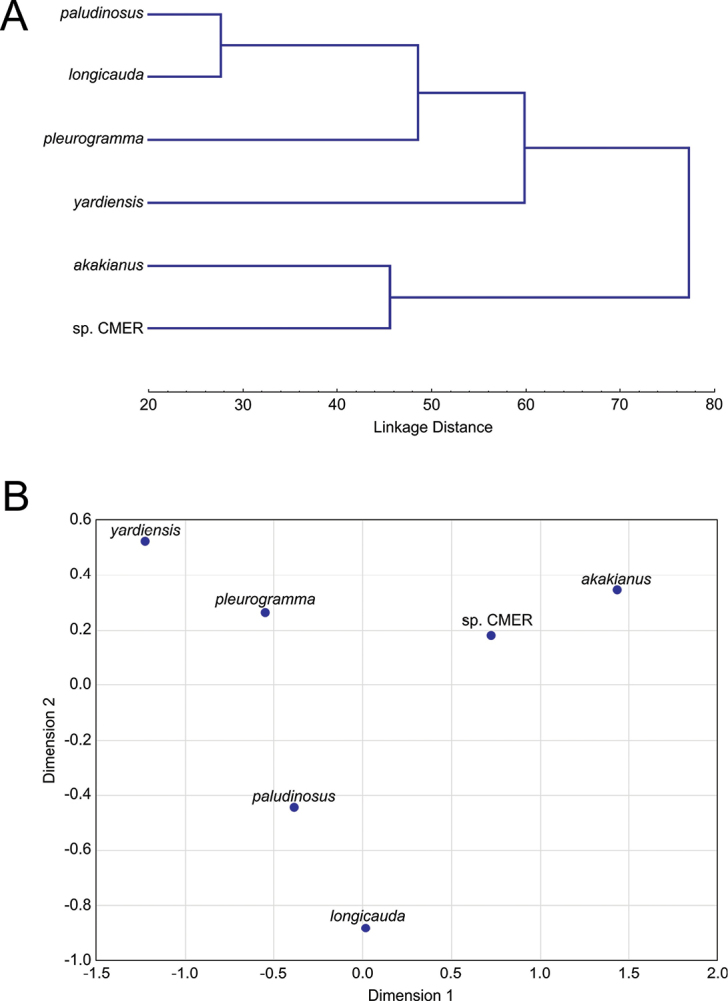
**A** CA and **B** MDS results for six samples based on means for 40 morphometric, 15 meristic and two coded qualitative characters (as in Tables 2–5). CMER referring to Central Main Ethiopian Rift as defined in the text.

**Figure 5.**
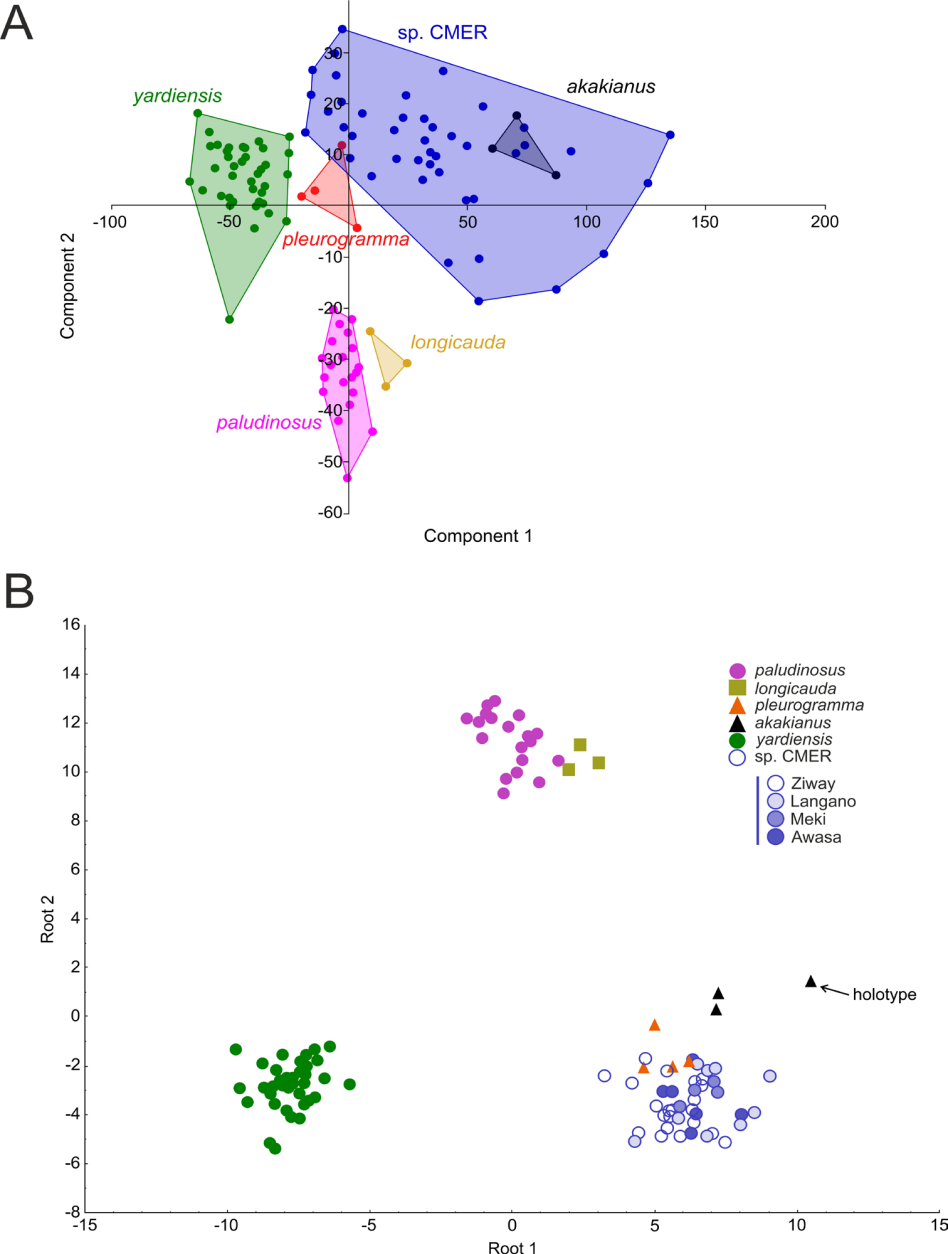
**A** PCA and **B** DFA results for six samples based on individual data. CMER referring to Central Main Ethiopian Rift as defined in the text.

The results of CA, MDS, PCA, and DFA (based on individual data per specimen) are given in Figs 4–5. For the analyses, 40 morphometric (proportional measurements), 16 meristic, and two coded characters were used. For routine statistics see Suppl. material 1, Tables S5–S10.

Based on the distribution (Fig. 1), the data for CO1 and cytb presented above as well as on primary morphological data that demonstrate a high level of similarity (Tables 3–5, Suppl. material 1, Table S3), we combined all samples from the CMER (Lake Ziway and its basin, Lake Langano and Lake Awasa) into one sample (Tables 3–5).

Both CA and MDS based on means per group (Fig. 4) clustered *E. paludinosus* and *E. longicauda* (both from the Zambezi drainage) together (Euclidean distance 0.597) while they are distinct from all Ethiopian congeners (0.725–1.984) though *E. pleurogramma* is closest to *E. paludinosus* in CA. Among the Ethiopian samples, the Lower Awash sample (see description of the new species) is closest to *E. pleurogramma* (0.724) and the specimens of *E. akakianus* are closest to the CMER group (the Meki River, Lake Ziway, Lake Langano and Lake Awasa).

As a further step, PCA and DFA were performed based on data per individual (Fig. 5). Based on the PCA, the most influential variables are length of posterior barbel (both % of horizontal eye diameter and % of HL) and length of anterior barbel (both % of horizontal eye diameter and % of HL). Statistics of factor coordinates of the variables can be seen in Suppl. material 1, Table S6. The syntypes of *E. pleurogramma* are separated but lay comparatively close to the Central Main Ethiopian Rift samples including *E. akakianus*. All Ethiopian samples are well separated from *E. paludinosus* and *E. longicauda*, which are morphologically very close (Fig. 5A, Suppl. material 1, Table S6).

The DFA (Fig. 5B) demonstrated a similar pattern of morphological affinities. Predicted classifications for *E. paludinosus*, *E. longicauda*, *E. pleurogramma*, *E. akakianus* and the samples from the Lower Awash and CMER were 100 % correct (Suppl. material 1, Table S8). DFA statistics: Wilks’ Lambda 0.00001, approx. F (170,357) = 19.832, p < 0.0000. Variables that contribute most for discrimination of the samples (Partial Lambda < 0.6) were the length of the lower non-serrated section of the last unbranched dorsal-fin ray (0.344), number of serrae on the last unbranched dorsal-fin ray (0.407), minimal caudal peduncle depth (two proportional measurements: % SL 0.531 and % caudal peduncle length 0.576), and caudal peduncle length (0.554).

The Lower Awash sample (described below as a new species) is morphologically the most distant from *E. longicauda*, *E. akakianus* and *E. paludinosus* (Squared Mahalanobis Distance equals 463.68, 373.59, and 275.90, respectively). The CMER samples are closer to *E. pleurogramma* (81.76) and *E. akakianus* (126.43) and the most distant from *E. longicauda* (388.89) being well separated also from the Lower Awash sample (206.79).

To summarise, in all statistical analyses 1) the Lower Awash sample is distinct from all Ethiopian congeners and the type series of *E. paludinosus* and *E. longicauda*; 2) the holotype of *E. akakianus* and two non-type specimens from the Akaki River are closest to (or imbedded into) the CMER group (Lake Langano, the Meki River, Lake Ziway and Lake Awasa); and 3) *E. paludinosus* and *E. longicauda* are morphologically closest taxa.

These results combined with the CO1 and cytb data provide a solid support to consider the Lower Awash River population of *Enteromius* as a distinct species described below.

#### Enteromius yardiensis sp. nov.

Taxon classificationAnimaliaCypriniformesCyprinidae

FE1AF1B9-DF2A-5E02-8927-D1941BF282ED

http://zoobank.org/444F1EDC-BA2C-4922-B8DE-0396EB6A343C

**Figure 6.**
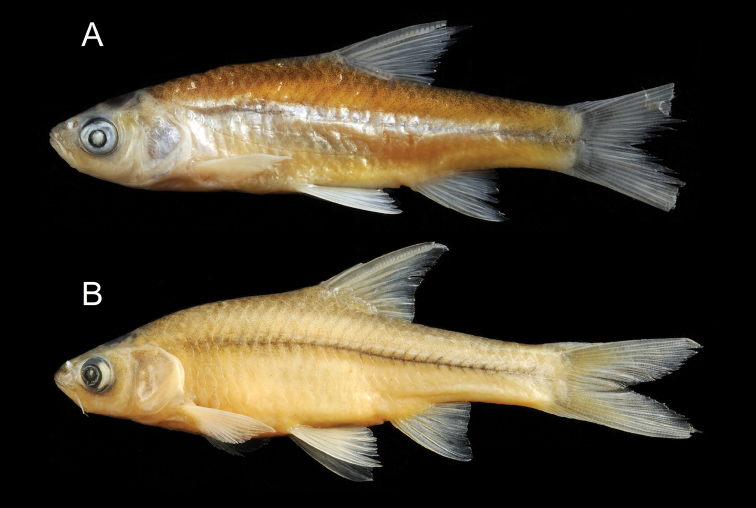
General appearance of *Enteromius yardiensis* sp. nov. **A** holotype, BMNH 2019.10.10.1, side channel of Awash R. at Kada Bada (site 2), 40.2 mm SL, The Trustees of the Natural History Museum, London **B** longest paratype, NMW 99642, Lake Gamari (site 5), 52.8 mm SL.

**Figure 7.**
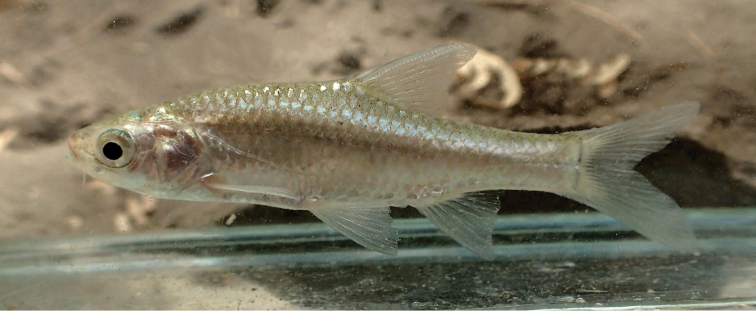
*Enteromius yardiensis* sp. nov. alive, paratype, NMW 99640, Awash R. at Aditu (site 3), 35.4 mm SL. Photograph by W. Graf.

**Figure 8.**
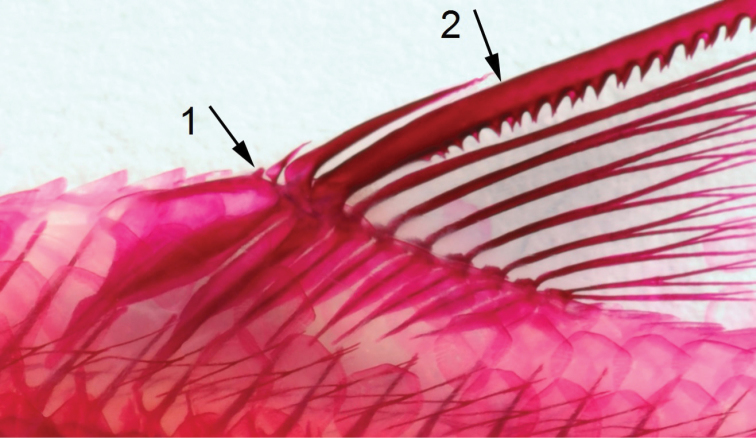
Dorsal fin in *Enteromius yardiensis* sp. nov., paratype, NMW 99259, side channel of Awash R. at Kada Bada (site 2), 37.6 mm SL, with four unbranched rays. Arrow 1 showing first unbranched dorsal-fin ray, arrow 2 showing relative positions of tip of penultimate (3^rd^) unbranched dorsal-fin ray and lowermost limit of serrated part of last (4^th^) unbranched dorsal-fin ray.

**Figure 9.**
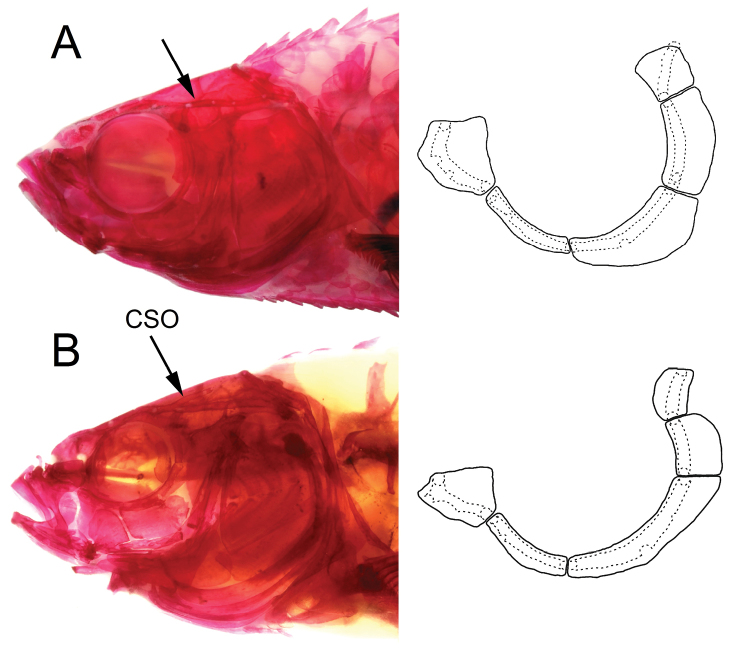
Alizarin-stained specimens showing cephalic sensory canals and infraorbitals in **A***Enteromius yardiensis* sp. nov. (same specimen as in Fig. 8) **B***Enteromius* sp. CMER, NMW 99237, Lake Ziway (site 8), 34.8 mm SL. Arrows showing part of frontal with no canal in **A** and medial branch of supraorbital canal (CSO) in **B**.

**Figure 10.**
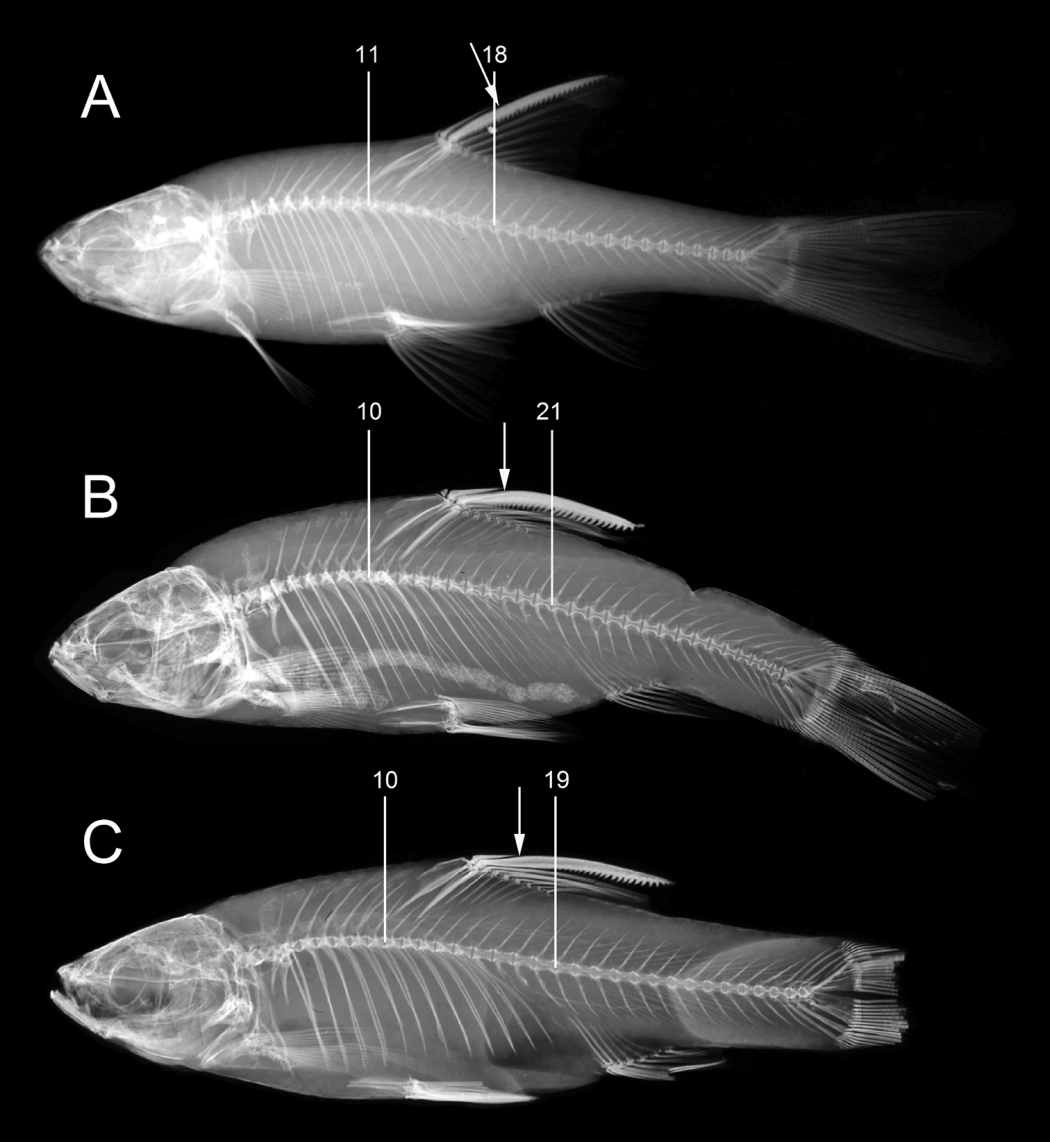
Axial skeletons of *Enteromius.***A***E. yardiensis* sp. nov. (same specimen as in Fig. 6B), 11 showing last predorsal abdominal vertebra and 18 last abdominal vertebra, total vertebrae 33:18+15 **B***E. akakianus*, holotype, BMNH 1908.1.20.85, Akaki R. (site 13), 66.0 mm SL, 10 showing last predorsal abdominal vertebra and 21 last abdominal vertebra, total vertebrae 37:21+16, The Trustees of the Natural History Museum, London **C***E. paludinosus*, paralectotype, BMNH 1861.3.10.6–7, Quellimane Mozambique, 50.0 mm SL, 10 showing last predorsal abdominal vertebra and 19 last abdominal vertebra, total vertebrae 34:19+15, The Trustees of the Natural History Museum, London. Arrows showing relative positions of tip of penultimate unbranched dorsal-fin ray and lowermost limit of serrated part of last unbranched dorsal-fin ray.

Figs 6 , 7 , 8 , 9A , 10A

##### Material examined.

**Holotype** (Fig. 6A). BMNH 2018.10.10.1, holotype, 40.2 mm SL (voucher specimen for CO1: MN747019), side channel of the Awash River at Kada Bada, north of the bridge on the way to Herto Bouri (site 2; 10°13'53"N, 40°34'43"E; 565 m a.s.l.), Afar Region, Ethiopia, 28.01.2018, coll. G.K. Englmaier, G. Tesfaye, P. Meulenbroek and H. Waidbacher.

**Paratypes**. BMNH 2018.10.10.2–4, 3, 35.6–38.8 mm SL, same date and locality as holotype. BMNH 2018.10.10.5–24, 20, 17.9–26.2 mm SL, same date and locality as holotype. NMW 99232, 1, 34.1 mm SL, same locality as holotype, 21.05.2018, coll. G.K. Englmaier and G. Tesfaye. NMW 99233, 18, 23.1–41.0 mm SL, same date and locality as NMW 99232. NMW 99234, 16, 24.9–31.2 mm SL, four voucher specimens for CO1 (MN747015, 29.5 mm SL; MN747016, 27.2 mm SL; MN747017, 24.3 mm SL; and MN747018, 22.8 mm SL), two vouchers for cytb (MN747022, 29.5 mm SL; and MN747023, 27.2 mm SL) and three C&S specimens in a separate jar (23.4–29.0 mm SL), all same date and locality as holotype. NMW 99235, 21, 17.4–24.1 mm SL, same date and locality as holotype. NMW 99259, 13, 24.4–33.4 mm SL, three C&S specimens in a separate jar (25.6–37.6 mm SL), same date and locality as NMW 99232. NMW 99487, 25, 20.4–42.2 mm SL, Awash River at Aditu [Adayitu], downstream of the bridge (site 3; 11°7'46"N, 40°45'52"E; 465 m a.s.l.), Afar Region, Ethiopia, 12.03.2019, coll. G.K. Englmaier, G. Tesfaye, P. Meulenbroek and H. Waidbacher. NMW 99488, 13, 20.9–32.6 mm SL, two voucher specimens for cytb (MN747024, 32.9 mm SL; and MN747025, 28.4 mm SL), same date and locality as NMW 99487. NMW 99493, 17, 26.2–33.8 mm SL, Awash River at Dubti (site 4; 11°41'50"N, 41°7'22"E; 375 m a.s.l.), Afar Region, Ethiopia, 13.03.2019, coll. G.K. Englmaier, G. Tesfaye, P. Meulenbroek and H. Waidbacher. NMW 99494, 8, 17.2–33.9 mm SL, two voucher specimens for cytb (MN747026, 41.0 mm SL; and MN747027, 42.8 mm SL) same date and locality as NMW 99493. NMW 99500, 4, 8.0–22.2 mm SL, western shore of Lake Gamari (site 5; 11°30'54"N, 41°38'57"E; 342 m a.s.l.), Afar Region, Ethiopia, 14.03.2019, coll. G.K. Englmaier, G. Tesfaye, P. Meulenbroek and H. Waidbacher. NMW 99501, 5, 8.9–23.2 mm SL, two voucher specimens for cytb (MN747028, 36.5 mm SL; and MN747029, 43.1 mm SL), same date and locality as NMW 99500. NMW 99639, 1, 28.7 mm SL, eastern shore of Lake Yardi (site 1; 10°14'41"N, 40°32'44"E; 565 m a.s.l.), Afar Region, Ethiopia, 21.05.2018, coll. G.K. Englmaier and G. Tesfaye. NMW 99640, 5, 30.3–42.9 mm SL, same date and locality as NMW 99487. NMW 99641, 8, 31.9–46.7 mm SL, same date and locality as NMW 99493. NMW 99642, 2, 25.6–52.9 mm SL, same date and locality as NMW 996500.

##### Diagnosis.

*Enteromius yardiensis* sp. nov. belongs to a phenotypic group characterised by small size and the last unbranched dorsal-fin ray thickened and serrated. The new species is readily distinguished from its Ethiopian congeners by three unique specialisations: the absence of the anterior barbel, the absence of the medial branch of the supraorbital cephalic canal and few, 1–3, commonly two, scale rows between lateral line and anus. It further differs by posterior barbel usually shorter than half eye diameter; eye large, its diameter 24–34 % HL; snout short and pointed; lateral line complete and strongly curved; scales in the lateral series 32–35; few transversal scale rows between lateral line and pelvic-fin base (1–3); scale rows between dorsal- and pelvic-fin origins 7–10; often four unbranched dorsal-fin rays; few branched pectoral-fin rays, commonly 12 or 13; 17 or 18 abdominal vertebrae; 10–12 predorsal abdominal vertebrae; and 6–9 vertebrae between first pterygiophores of dorsal and anal fins.

##### Description.

Morphometric and meristic characters of the holotype are in Tables 2, 4–5. The general appearance of *E. yardiensis* is shown in Figs 6, 7, dorsal fin in Fig. 8, sensory canals in Fig. 9A, axial skeleton in Fig. 10A and relative measurements of the holotype and paratypes are given in Table 2 and Suppl. material 1, Table S3. Variations in 14 meristic characters and numerically coded types of posterior barbel length are provided in Table 4, and data on the number of serrae on last unbranched dorsal-fin ray in Table 5.

**Table 2.** Morphometrics of *Enteromius yardiensis* sp. nov.; holotype, BMNH 2018.10.10.1 (in separate column); paratypes, BMNH 2018.10.10.2–4, NMW 99232, NMW 99233, NMW 99639, NMW 99640, NMW 99641, NMW 99642.

Measurements	BMNH 2018.10.10.2–4, holotype	holotype and paratypes				
		n	Min	Max	Mean	S.D.
SL, mm	52.80	69	17.9	52.80	30.5	7.1
Body depth at pelvic-fin origin (% SL)	28.4	39	23.5	28.4	26.1	1.4
Minimum caudal-peduncle depth (% SL)	12.2	39	10.4	12.9	11.8	0.5
Minimum caudal-peduncle depth (% caudal-peduncle length)	54.0	39	45.3	59.1	52.7	3.1
Maximum caudal-peduncle depth (% SL)	14.8	39	11.8	15.2	14.0	0.8
Maximum caudal-peduncle depth (% caudal-peduncle length)	65.7	39	50.1	68.7	62.0	4.0
Predorsal length (% SL)	55.5	39	53.0	58.1	55.7	1.4
Prepelvic length (% SL)	48.3	39	47.6	51.1	49.3	0.9
Preanal length (% SL)	67.4	39	67.4	71.6	69.8	0.9
Pectoral – pelvic Euclidean distance (% SL)	22.6	39	19.7	23.3	21.2	1.0
Pelvic – anal Euclidean distance (% SL)	21.3	39	18.8	23.2	21.3	1.0
Caudal-peduncle length (% SL)	22.6	39	21.0	24.5	22.5	1.0
Dorsal-fin depth (% SL)	27.6	39	22.3	31.3	28.0	1.6
Dorsal-fin depth (% HL)	100.4	39	76.9	113.2	99.8	7.0
Length of last unbranched dorsal-fin ray excluding the segmented part (% SL)	24.1	39	18.3	24.2	21.6	1.6
Length of last unbranched dorsal-fin ray including the segmented part (% SL)	0.0	16	23.4	26.7	25.3	1.1
Anal-fin depth (% SL)	18.4	39	16.7	20.5	19.1	0.8
Pectoral-fin length (% SL)	20.2	39	16.4	20.8	19.3	0.9
Pectoral-fin length (% pectoral – pelvic Euclidean distance)	89.4	39	81.7	101.7	90.9	5.1
Pelvic-fin length (% SL)	19.9	39	17.7	21.0	19.3	0.9
Pelvic-fin length (% pelvic – anal Euclidean distance)	93.2	39	81.8	101.6	90.6	5.1
Pelvic-splint length (% pelvic-fin length)	21.4	39	13.2	31.8	21.6	3.2
Head length (% SL)	27.4	39	25.5	31.5	28.1	1.5
Head length (% body depth at pelvic-fin origin)	96.5	39	94.5	131.5	108.1	7.4
Head depth at nape (% SL)	18.8	39	17.7	21.8	19.5	1.0
Head depth at nape (% HL)	68.6	39	62.9	75.9	69.4	3.1
Head depth at posterior eye margin (% SL)	16.2	39	15.8	20.5	17.7	1.1
Head depth at posterior eye margin (% HL)	59.1	39	57.9	72.6	63.1	3.4
Head width (% SL)	14.8	39	12.6	17.4	14.9	1.3
Head width (% HL)	54.1	39	46.2	65.3	53.0	3.4
Snout length (% SL)	7.3	39	6.1	7.7	6.7	0.4
Snout length (% HL)	26.6	39	20.8	26.7	24.0	1.5
Eye horizontal diameter (% SL)	6.4	39	6.4	10.1	8.1	0.9
Eye horizontal diameter (% HL)	23.5	39	23.5	33.6	28.8	2.2
Eye horizontal diameter (% interorbital width with skin fold)	63.3	39	63.3	93.1	78.1	6.6
Interorbital width with skin fold (% SL)	10.2	39	9.3	11.8	10.4	0.6
Interorbital width with skin fold (% HL)	37.1	39	33.2	40.4	36.9	1.5
Interorbital width between frontal margins (% SL)	6.6	39	4.9	8.7	6.2	0.9
Interorbital width between frontal margins (% HL)	24.2	39	17.1	29.4	22.2	3.8
Lower-jaw length (% SL)	9.6	39	8.1	10.1	9.2	0.4
Lower-jaw length (% HL)	35.0	39	29.6	36.1	32.9	1.5
Lower-jaw length (% interorbital width with skin fold)	94.3	39	79.4	97.2	89.2	4.8
Lower-jaw length (% operculum depth)	85.9	39	71.2	91.2	80.5	4.9
Lower-jaw length (% maximum cranium width)	81.6	39	66.5	81.6	74.6	3.5
Operculum depth (% SL)	11.2	39	10.1	13.4	11.5	0.8
Operculum depth (% HL)	40.8	39	37.9	44.5	40.9	1.5
Cranial-roof length (% SL)	17.6	39	15.9	21.6	18.4	1.4
Cranial-roof length (% HL)	64.2	39	55.7	71.6	65.6	3.4
Maximum cranium width (% cranial-roof length)	66.7	39	59.8	78.8	67.4	4.3
Anterior barbel length (% HL)	0.0	39	0.0	0.0	0.0	0.0
Anterior barbel length (% eye horizontal diameter)	0.0	39	0.0	0.0	0.0	0.0
Posterior barbel length (% HL)	14.0	39	4.2	17.8	11.5	3.0
Posterior barbel length (% eye horizontal diameter)	59.5	39	13.9	65.0	40.4	12.3
Length of unsegmented part from x-ray (% dorsal-fin depth)	86.9	62	76.3	95.1	88.3	4.9
Length of lower non-serrated part from x-ray (% dorsal-fin depth)	11.9	62	11.9	32.9	20.5	4.9
Length of upper serrated part from x-ray (% dorsal-fin depth)	75.0	62	49.8	77.5	67.7	5.7
length of lower non-serrated part from x-ray (% length of unsegmented part)	13.7	62	13.7	38.0	23.2	5.2
length of upper serrated part from x-ray (% length of unsegmented part)	86.3	62	62.0	86.3	76.7	5.3

**Table 3.** Morphometric data of *Enteromius akakianus*, *E. pleurogramma*, *Enteromius* sp. CMER, *E. paludinosus*, and *E. longicauda*; blank spaces indicating missing data. CMER referring to Central Main Ethiopian Rift as defined in text. Information per specimen as in Table 1.

Measurements	*E. akakianus*, holotype	*E. akakianus*	*E. akakianus*	*E. pleurogramma*					*Enteromius* sp. CMER					*E. paludinosus*					*E. longicauda*				
				n	Min	Max	Mean	S.D.	n	Min	Max	Mean	S.D.	n	Min	Max	Mean	S.D.	n	Min	Max	Mean	S.D.
SL, mm	66.0	88.5	67.8	4	28.5	35.4	31.7	3.0	47	31.4	70.4	45.8	9.3	20	35.5	70.5	51.1	11.3	3	61.1	67.0	64.6	3.1
Body depth at pelvic-fin origin (% SL)	30.2	28.4	26.0	4	23.1	26.4	24.4	1.4	43	22.3	28.8	25.5	1.6	20	25.1	30.8	27.8	1.8	3	26.3	28.1	27.2	0.9
Minimum caudal-peduncle depth (% SL)	14.0	12.0	12.6	4	10.9	12.9	12.0	0.8	42	8.4	13.6	12.1	1.0	20	12.6	14.7	13.5	0.5	3	11.6	12.1	11.9	0.3
Minimum caudal-peduncle depth (% caudal-peduncle length)	66.0	56.8	57.8	4	46.6	59.8	52.0	5.6	42	35.6	65.1	54.4	5.2	20	48.0	62.2	55.2	3.7	3	41.6	45.2	43.8	1.9
Maximum caudal-peduncle depth (% SL)	14.7	15.1	12.9	4	13.2	14.0	13.6	0.3	42	10.2	16.6	14.0	1.2	5	14.9	16.6	15.8	0.7					
Maximum caudal-peduncle depth (% caudal-peduncle length)	69.1	71.7	59.3	4	56.6	63.8	59.3	3.2	42	43.0	74.4	62.7	6.2	5	62.7	70.1	66.0	3.3					
Predorsal length (% SL)	53.5	54.5	54.5	4	51.7	54.3	53.1	1.1	41	49.3	56.0	52.6	1.5	20	50.9	56.3	53.0	1.5	3	49.4	54.2	51.7	2.4
Prepelvic length (% SL)	51.9	49.7	49.8	4	50.6	53.0	51.9	1.0	42	46.2	52.7	50.2	1.4	20	45.1	51.2	47.8	1.8	3	45.0	46.5	45.9	0.8
Preanal length (% SL)	72.6	70.9	72.6	4	73.5	74.4	74.1	0.4	42	68.1	74.5	71.9	1.4	20	65.6	74.7	71.0	2.0	3	66.6	68.3	67.4	0.9
Pectoral – pelvic Euclidean distance (% SL)	25.4	21.5	21.6	4	20.3	23.9	22.4	1.5	43	20.0	25.7	22.4	1.3	5	20.6	22.9	21.4	0.9					
Pelvic – anal Euclidean distance (% SL)	23.0	24.2	24.1	4	21.7	23.7	22.9	0.9	42	20.1	27.8	22.9	1.5	5	24.6	26.4	25.5	0.7					
Caudal-peduncle length (% SL)	21.3	21.1	21.7	4	21.6	23.7	23.0	1.0	42	20.5	25.1	22.4	1.0	20	22.2	26.8	24.5	1.4	3	25.6	29.1	27.2	1.8
Dorsal-fin depth (% SL)	28.7	23.4	25.2	4	21.0	27.7	24.6	2.8	40	19.9	29.8	25.0	2.2	19	23.2	30.5	26.9	1.8	3	21.9	25.5	23.3	1.9
Dorsal-fin depth (% HL)	108.5	86.2	95.3	4	84.4	100.5	90.5	7.1	40	71.7	108.2	89.8	7.5	19	94.3	119.3	103.4	5.6	3	91.2	106.5	96.4	8.8
Length of last unbranched dorsal-fin ray excluding the segmented part (% SL)	21.8	19.7	18.5	3	17.1	18.9	17.8	0.9	33	13.5	24.1	19.1	2.3	17	18.8	24.5	21.6	1.7	3	18.8	22.0	19.9	1.8
Length of last unbranched dorsal-fin ray including the segmented part (% SL)									23	18.0	26.7	22.6	2.1										
Anal-fin depth (% SL)	17.0	16.1	17.8	4	16.2	18.0	17.0	0.8	42	13.0	19.0	16.7	1.1	19	16.0	20.0	17.6	1.0	3	15.4	16.3	15.9	0.4
Pectoral-fin length (% SL)	20.1	16.4	19.3	4	16.5	18.3	17.1	0.8	43	15.0	20.8	18.1	1.2	20	18.4	21.7	19.5	0.9	3	17.9	18.8	18.4	0.4
Pectoral-fin length (% pectoral – pelvic Euclidean distance)	78.9	76.2	89.3	4	69.5	84.1	76.6	6.7	43	68.0	95.1	80.9	6.3	5	80.4	93.4	88.1	5.1					
Pelvic-fin length (% SL)	18.4	16.7	19.7	4	13.5	15.8	14.8	1.0	43	15.0	19.5	17.1	0.8	20	16.2	22.9	19.3	1.5	3	18.0	18.2	18.1	0.1
Pelvic-fin length (% pelvic – anal Euclidean distance)	79.7	69.0	82.0	4	59.1	68.8	64.7	4.3	42	53.9	85.7	75.2	6.2	5	63.1	76.7	69.3	5.4					
Pelvic-splint length (% pelvic-fin length)	24.0	15.0	18.8	4	23.2	35.5	29.6	5.5	43	12.8	34.9	23.4	4.1	5	17.9	26.3	21.3	3.2					
Head length (% SL)	26.5	27.2	26.5	4	24.9	28.6	27.2	1.6	47	25.9	31.1	28.0	1.3	20	24.0	27.5	26.0	0.9	3	23.9	24.4	24.2	0.3
Head length (% body depth at pelvic-fin origin)	87.6	95.7	101.7	4	107.7	116.6	111.6	4.2	43	93.7	122.7	109.2	7.0	20	81.5	109.5	94.1	7.5	3	85.5	92.9	88.7	3.8
Head depth at nape (% SL)	18.4	17.9	17.4	4	17.7	19.9	19.0	0.9	46	17.2	21.4	19.5	0.9	5	18.2	20.1	18.9	0.8					
Head depth at nape (% HL)	69.5	65.9	65.8	4	69.0	71.3	70.0	1.0	46	63.7	77.0	69.5	2.9	5	69.2	74.5	71.2	2.0					
Head depth at posterior eye margin (% SL)	17.1	15.9	16.5	4	15.4	18.9	17.4	1.4	42	15.3	19.6	17.6	1.0	20	15.2	17.6	16.2	0.8	3	14.1	15.0	14.5	0.5
Head depth at posterior eye margin (% HL)	64.6	58.5	62.2	4	62.1	66.1	63.7	1.9	42	58.5	69.5	63.1	2.6	20	58.0	65.4	62.4	2.0	3	57.6	62.6	60.0	2.5
Head width (% SL)	13.8	12.4	13.1	4	11.0	13.2	12.1	1.0	42	12.2	16.8	14.3	1.2	20	12.0	14.9	13.5	0.9	3	12.7	13.4	13.1	0.4
Head width (% HL)	51.9	45.6	49.6	4	41.3	46.1	44.4	2.2	42	44.5	60.0	51.4	4.0	20	45.6	57.5	51.9	3.4	3	52.6	55.5	54.3	1.5
Snout length (% SL)	6.9	6.8	6.9	4	6.1	6.9	6.7	0.4	42	6.0	8.1	7.1	0.5	20	6.1	8.0	6.9	0.5	3	6.1	6.8	6.5	0.3
Snout length (% HL)	26.0	25.1	25.9	4	23.7	25.0	24.5	0.6	42	22.1	28.7	25.4	1.6	20	24.2	30.4	26.6	1.7	3	25.5	27.6	26.8	1.1
Eye horizontal diameter (% SL)	6.7	6.1	6.7	4	7.6	8.0	7.7	0.2	42	5.4	9.0	7.1	0.8	20	6.3	7.8	6.9	0.4	3	6.3	6.6	6.5	0.1
Eye horizontal diameter (% HL)	25.2	22.4	25.4	4	26.5	30.4	28.5	1.7	42	19.7	32.7	25.5	2.7	20	23.9	30.4	26.6	1.6	3	26.0	27.6	26.8	0.8
Eye horizontal diameter (% interorbital width with skin fold)	76.4	70.9	76.7	4	81.2	95.0	91.3	6.7	42	50.5	108.5	74.2	11.3	20	63.1	83.5	72.3	5.2	3	65.4	70.2	67.5	2.4
Interorbital width with skin fold (% SL)	8.7	8.6	8.8	4	8.0	9.3	8.5	0.6	42	7.7	12.0	9.7	0.9	20	8.8	10.4	9.6	0.5	3	9.4	9.7	9.6	0.2
Interorbital width with skin fold (% HL)	33.0	31.5	33.2	4	29.5	32.7	31.3	1.4	42	28.7	44.0	34.7	2.9	20	33.9	40.1	36.9	1.5	3	39.3	40.0	39.7	0.4
Interorbital width between frontal margins (% SL)	6.4	6.6	6.3	4	6.3	7.5	6.8	0.6	46	3.4	7.1	5.6	0.9	20	6.3	8.1	7.3	0.4	3	5.9	6.5	6.2	0.3
Interorbital width between frontal margins (% HL)	24.3	24.3	23.8	4	22.9	26.2	24.8	1.4	46	12.0	25.3	20.1	3.2	20	23.9	31.2	28.1	1.7	3	24.3	27.0	25.8	1.4
Lower-jaw length (% SL)	9.1	8.8	8.2	4	9.0	9.8	9.4	0.4	47	7.8	9.9	9.1	0.4	5	9.0	9.6	9.2	0.2					
Lower-jaw length (% HL)	34.4	32.5	31.1	4	33.0	36.1	34.7	1.4	47	28.8	36.1	32.7	1.8	5	33.9	36.0	34.7	1.0					
Lower-jaw length (% interorbital width with skin fold)	104.2	103.2	93.8	4	105.3	114.7	111.1	4.1	42	69.6	121.6	95.4	11.2	5	89.9	97.4	92.7	2.8					
Lower-jaw length (% operculum depth)	84.1	87.1	78.0	4	80.0	92.2	87.9	5.5	47	70.1	93.8	80.7	5.4	5	82.1	86.5	83.8	1.7					
Lower-jaw length (% maximum cranium width)	73.8	78.5	71.9	4	76.6	80.4	78.5	1.7	46	65.0	86.4	75.0	5.0	5	72.4	80.3	76.7	3.8					
Operculum depth (% SL)	10.8	10.2	10.6	4	9.8	11.4	10.8	0.7	47	9.9	13.2	11.4	0.7	5	10.7	11.5	11.0	0.3					
Operculum depth (% HL)	40.9	37.3	39.9	4	38.4	41.2	39.6	1.2	47	36.3	43.9	40.6	1.6	5	40.7	42.8	41.4	0.8					
Cranial-roof length (% SL)	15.8	16.6	16.2	4	18.2	19.8	19.2	0.8	46	16.4	21.0	17.8	1.0	5	15.5	16.5	16.2	0.4					
Cranial-roof length (% HL)	59.7	61.1	61.2	4	68.0	73.3	70.5	2.5	46	56.5	69.9	63.6	3.1	5	58.9	62.5	60.9	1.4					
Maximum cranium width (% cranial-roof length)	78.0	67.8	70.7	4	61.2	64.1	62.7	1.2	46	58.5	78.2	68.7	4.3	5	72.5	77.8	74.3	2.2					
Anterior barbel length (% HL)	21.9	16.0	17.5	4	8.4	10.0	9.2	0.7	46	4.6	24.0	11.9	4.6	20	4.0	11.6	7.3	2.1	3	10.9	13.6	11.9	1.5
Anterior barbel length (% eye horizontal diameter)	86.6	71.4	68.9	4	31.3	33.8	32.4	1.2	42	16.6	109.7	49.5	22.4	20	15.6	45.4	27.6	7.9	3	40.7	49.2	44.2	4.4
Posterior barbel length (% HL)	33.8	27.6	29.3	4	14.2	19.7	17.2	2.5	46	15.4	38.7	24.4	5.3	20	14.2	25.7	19.7	2.7	3	18.1	23.7	21.0	2.8
Posterior barbel length (% eye horizontal diameter)	134.0	123.6	115.1	4	46.7	74.1	61.0	12.1	42	54.1	177.3	99.6	29.2	5	62.2	77.9	69.8	6.5	3	69.8	85.9	78.4	8.1
length of unsegmented part from x-ray (% dorsal-fin depth)	88.7	92.9	92.5	3	80.1	90.9	86.8	5.8	35	73.1	96.1	85.1	6.7	17	83.3	96.5	91.4	4.5	3	92.4	93.4	93.0	0.5
length of lower non-serrated part from x-ray (% dorsal-fin depth)	15.0	11.5	17.2	3	24.2	29.8	26.5	2.9	35	10.4	25.4	17.4	3.3	17	32.8	43.1	37.4	2.9	3	32.6	38.2	35.2	2.8
length of upper serrated part from x-ray (% dorsal-fin depth)	73.7	81.5	75.3	3	54.5	65.1	60.2	5.4	35	50.8	81.2	67.7	7.9	17	45.1	61.7	54.0	5.0	3	55.1	59.7	57.8	2.4
length of lower non-serrated part from x-ray (% length of unsegmented part)	16.9	12.3	18.6	3	27.1	32.8	30.6	3.1	35	12.7	31.0	20.6	4.5	17	35.9	46.6	41.0	3.4	3	35.3	40.9	37.8	2.9
length of upper serrated part from x-ray (% length of unsegmented part)	83.1	87.7	81.4	3	67.2	72.9	69.4	3.1	35	69.0	87.3	79.4	4.5	17	53.4	64.1	59.0	3.4	3	59.1	64.7	62.2	2.9

**Table 4.** Frequencies of occurrence of meristic character states and coded length of anterior- and posterior barbels in *Enteromius yardiensis* sp. nov., *E. akakianus*, *E. pleurogramma*, *Enteromius* sp. CMER, *E. paludinosus*, and *E. longicauda*. Values with * indicating counts found in holotypes and lectotypes. Numbers in squared brackets refer to mean±SD; blank spaces indicate missing data. CMER referring to Central Main Ethiopian Rift as defined in text. Information per specimen as in Table 1.

**Character states**	***E. yardiensis***	***E. akakianus***	***E. pleurogramma***	***Enteromius* sp. CMER**	***E. paludinosus***	***E. longicauda***
Number of unbranched dorsal-fin rays	3(47), 4*(22) [3.3±0.5]	3*(2), 4(1) [3.3±0.6]	3(1), 4(3) [3.8±0.5]	2(1), 3(38), 4(7) [3.1±0.4]	2(3), 3*(14), 4(3) [3.0±0.6]	3*(2), 4(1) [3.3±0.6]
Number of branched pelvic-fin rays	7*(35), 8(4) [7.1±0.3]	7*(1), 8(2) [7.7±0.6]	8(4) [8.0±0.0]	6(1), 7(3), 8(38), 9(5) [8.0±0.5]	7(1), 8*(18), 9(1) [8.0±0.3]	7(1), 8*(2) [7.7±0.6]
Number of branched pectoral-fin rays	12*(11), 13(23), 14(5) [12.8±0.6]	16*(3) [16.0±0.0]	13(1), 14(1), 15(2) [14.3±1.0]	12(1), 14(5), 15(25), 16(16) [15.2±0.8]	13(2), 14*(9), 15(9) [14.4±0.7]	15*(2), 16(1) [15.3±0.6]
Total number of vertebrae	33*(53), 34(16) [33.2±0.4]	35(1), 36(1), 37*(1) [36.0±1.0]	35(4) [35.0±0.0]	34(2), 35(29), 36(13), 37(2) [35.3±0.6]	34*(17), 35(3) [34.2±0.4]	35(2), 36*(1) [35.3±0.6]
Number of abdominal vertebrae	17(18), 18*(50) [17.7±0.4]	20(2), 21*(1) [20.3±0.6]	19(1), 20(3) [19.8±0.5]	19(14), 20(27) [19.7±0.5]	18*(18), 19(2) [18.1±0.3]	19*(3) [19.0±0.0]
Number of caudal vertebrae	15*(35), 16(32), 17(1) [15.5±0.5]	15(1), 16*(2) [15.7±0.6]	15(3), 16(1) [15.3±0.5]	14(1), 15(25), 16(15), 17(1) [15.4±0.6]	15(1), 16*(17), 17(2) [16.1±0.4]	16(2), 17*(1) [16.3±0.6]
Number of predorsal abdominal vertebrae	10(8), 11*(60), 12(1) [10.9±0.3]	10*(2), 11(1) [10.3±0.6]	11(3), 12(1) [11.3±0.5]	9(1), 10(20), 11(23), 12(1) [10.5±0.6]	9(1), 10*(19) [10.0±0.2]	10*(3) [10.0±0.0]
Number of preanal caudal vertebrae	0*(39), 1(19), 2(1) [0.4±0.5]	0*(2), 1(1) [0.3±0.6]	0(3), 1(1) [0.3±0.5]	0(24), 1(21), 2(1) [0.5±0.5]	0*(19), 1(1) [0.1±0.2]	0*(3) [0.0±0.0]
Number of vertebrae between first pterygiophores of dorsal and anal fins	6(5), 7*(52), 8(11), 9(1) [7.1±0.5]	10(2), 11*(1) [10.3±0.6]	8(1), 9(3) [8.8±0.5]	8(1), 9(11), 10(29), 11(2), 12(1) [9.8±0.7]	8*(15), 9(5) [8.3±0.4]	8(1), 9*(2) [8.7±0.6]
Total number of lateral-series scales	32(4), 33(23), 34*(10), 35(2) [33.3±0.7]	36(2), 37*(1) [36.3±0.6]	34(1), 35(2), 36(1) [35.0±0.8]	34(6), 35(17), 36(16), 37(3) [35.4±0.8]	33*(5), 34(7), 35(5), 36(3) [34.3±1.0]	35(1), 36*(1), 37(1) [36.0±1.0]
Number of lateral-series scales to posterior margin of hypurals	31(7), 32(19), 33*(9), 34(4) [32.3±0.9]	35*(2), 36(1) [35.3±0.6]	32(1), 34(3) [33.5±1.0]	32(1), 33(9), 34(11), 35(17), 36(4) [34.3±1.0]	32*(8), 33(5), 34(7) [33.0±0.9]	34*(2), 35(1) [34.3±0.6]
Number of scale rows between lateral line – dorsal-fin origin	6(28), 7*(11) [6.3±0.5]	6*(3) [6.0±0.0]	6(3), 7(1) [6.3±0.5]	5(1), 6(35), 7(6) [6.1±0.4]	6*(7), 7(13) [6.7±0.5]	7*(3) [7.0±0.0]
Number of scale rows between lateral line – pelvic fin origin	1(1), 2*(30), 3(8) [2.2±0.5]	3(2), 4*(1) [3.3±0.6]	4(4) [4.0±0.0]	3(4), 4(37) 5(1) [3.9±0.3]	3(3), 4*(17) [3.9±0.4]	3(1), 4*(2) [3.7±0.6]
Number of scale rows between lateral line – anus	1(1), 2*(33), 3(5) [2.1±0.4]	4(2), 5*(1) [4.3±0.6]	4(2), 5(2) [4.5±0.6]	3(1), 4(35), 5(6) [4.1±0.4]	4*(16), 5(4) [4.2±0.4]	4*(3) [4.0±0.0]
Anterior barbel coded length; note that the character is not applicable for *E. yardiensis* sp. nov. with anterior barbel absent in all specimens	absent	2*(3)	1(4)	1(36), 2(10)	1*(20)	1*(3)
Posterior barbel coded length	1(7), 2*(32)	3(2), 4*(1)	2(3), 3(1)	2(9), 3(35), 4(2)	2*(17), 3(3)	2*(3)

Longest examined specimen 52.8 mm SL (NMW 99642, Fig. 6B). Body markedly compressed. Dorsal head profile slightly concave, its transition to back smooth, slight nuchal hump only present in few larger individuals (more than 35 mm SL). Head commonly longer than body depth at pelvic-fin origin.

In most specimens, predorsal back outline steeply rising to dorsal-fin origin. Postdorsal back outline slightly convex or straight to end of caudal peduncle. Head depth at nape not exceeding HL. Snout pointed and relatively short, its length not exceeding eye horizontal diameter. Mouth terminal, tip of mouth cleft on about level of middle of eye, mouth cleft straight. Posterior barbel short (coded length 2), shorter than half eye diameter. Anterior barbel absent in all specimens (17.9–52.8 mm SL) but foramen for its nerve present in maxillary (examined in six C&S specimens). Eye large, its horizontal diameter greater than snout length but shorter than lower jaw length. Eye diameter negatively correlated with SL (R = -0.72 Spearman’s rank correlation, *N* = 39). Interorbital width is commonly less than eye horizontal diameter.

Dorsal fin with three or four unbranched and eight branched rays. Last unbranched ray moderately thickened and densely serrated. Serration extending over more than 76 % of ray non-segmented part (range 62–86 %) and lower non-serrated part relatively short and not strongly thickened (Table 2, Fig. 8). Number of serrae ranging from 10 (< 25 mm SL) to 27 (> 50 mm SL) and positively correlated with size (*R* = 0.96, Spearman's rank correlation). In case of four unbranched rays (in 32 % of examined specimens, Table 4), first ray often small and only visible in radiographs or in C&S individuals (Fig. 8). Predorsal body long and dorsal-fin origin placed behind origin of pelvic fin. Dorsal-fin depth slightly shorter than HL. Anal fin with three unbranched and six branched rays, reaching to about middle of caudal peduncle. Pelvic fin with one unbranched and commonly seven branched rays (Table 3), commonly reaching behind anus and rarely to anal-fin origin. Pelvic splint variable in length but relatively short (19–32 % of pelvic-fin length). Pectoral fin with one unbranched and commonly 12 or 13 branched rays (Table 4), commonly not reaching pelvic-fin origin. Pectoral, pelvic and anal fins of about similar length (Table 2). Caudal fin forked with 2+17 principal rays (commonly eight in upper lobe and seven in lower lobe). Upper procurrent rays 7 (11), 8 (17) or 9 (1), lower procurrent rays 6 (2), 7 (25) or 8 (2).

Lateral line complete (in all specimens with well-preserved scales) and clearly downwardly curved on the body going along midline on posterior half of caudal peduncle. Total lateral series with 32–35, commonly 33, scales (Table 4). Circumpeduncular scale rows 12–14 (mode 13 (6), *N* = 11). Back, belly and chest fully scaled. Short axillary scale present at pelvic-fin base.

Five infraorbital bones (io) but io4 often fragmented into two. Bones io3 to io5 wide, covering most surface of cheek in front of preoperculum; io5 as wide as deep or wider as deep (in larger specimens, Fig. 9A).

Supraorbital canal complete lacking medial branch of supraorbital canal in all specimens (Fig. 9A). Infraorbital and supratemporal canals complete. Preoperculo-mandibular canal complete between lower jaw and preoperculum and not communicating with postocular commissure of infraorbital canal; preopercular section of this canal terminating at upper margin of preoperculum or continuing into suprapreopercular segment fused to antedorsal process of operculum terminating by free pore over its upper margin or somewhat below.

Total vertebrae few, 33 (most commonly, Fig. 10A) or 34; other vertebral counts given in Table 4. Supraneurals 5–6, first three or four square-shaped and two or three in front of dorsal fin deeper and elongated.

Gill rakers in outer row of first gill arch 10 (5), 11 (4) or 12 (1), with eight or nine on lower limb and two on upper limb. Pharyngeal teeth thin and slightly hooked, not serrated, 2.3.5–5.3.2.

In four examined specimens (23.4–29.1 mm SL), length of digestive tract (not stretched) about 82–107 % of SL. Intestine folded in simple loop before reaching anus.

Mature females were observed during mid of dry season at sizes less than 40 mm SL (36.7–39.4 mm SL, *N* = 4). Early stage of maturation (the developing phase of the reproductive cycle according to Brown-Peterson et al. (2011)) in females was found between 28.9–29.5 mm SL (*N* = 2).

##### Colouration.

In life (Fig. 7), overall silvery, with greenish brown back. Greenish iridescence especially at nape and upper eye. Fins pale, base of pectoral, pelvic and anal fins sometimes faintly pinkish, caudal-fin base brownish. No stripe or blotches. Most ethanol-preserved specimens (Fig. 6A) overall silvery with much shine at midline, ventral and opercular regions. Other body parts brownish orange or silvery grey. Fins pale, base of caudal fin and, often, anterior part of dorsal-fin base ash-grey. Formalin (initial fixation) and later transferred to 75 % ethanol (Fig. 6B) specimens creamy to yellowish, formalin-deposited whitish. Back greyish brown, posterior head brownish black. Narrow black mid-lateral stripe usually of increasing intensity at caudal peduncle but not reaching caudal-fin base. Ventral body (especially bases of pectoral and anal fins) often yellowish. Fins pale, anterior part of dorsal-fin base brownish. Sparse melanophores on rays of dorsal, caudal and anal fins.

##### Distribution and habitat.

The new species was found so far only in the Lower Awash River and interconnected lakes (Fig. 1). The altitude ranges from 565 m a.s.l. (10°14'41"N, 40°32'44"E) to 342 m a.s.l. (11°30'54"N, 41°38'57"E). This wetland area is a part of the extensive Afar lowland which is a geological depression caused by the Afar Triple Junction, connected in the south to the north-eastern segment of the Main Ethiopian Rift (Beyene and Abdelsalam 2005).

**Figure 11.**
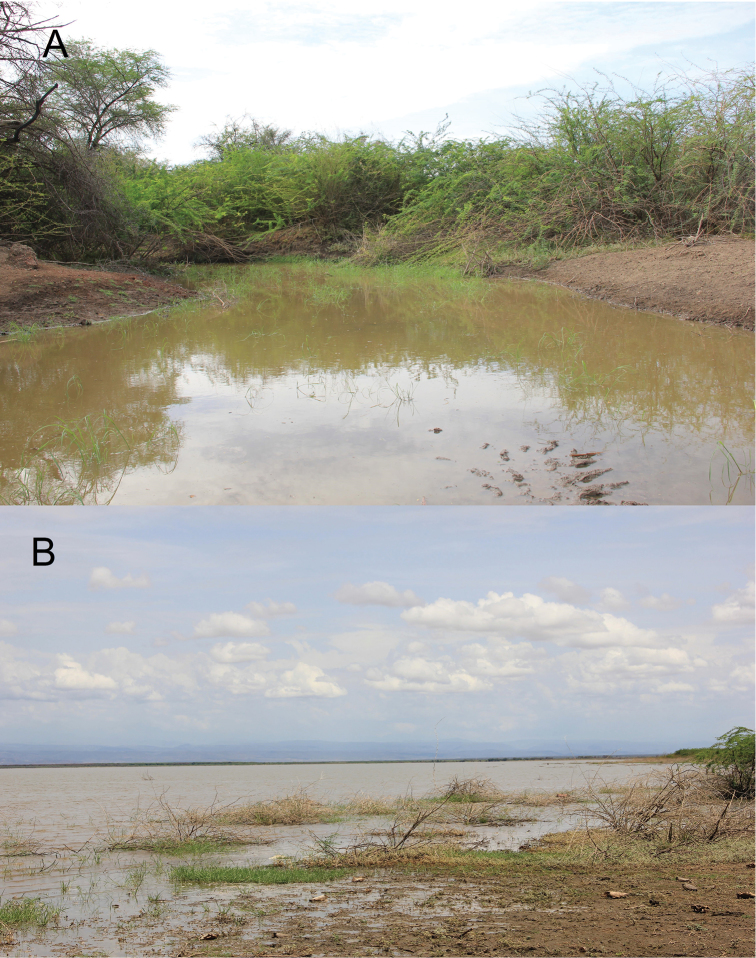
Habitat of *Enteromius yardiensis* sp. nov. **A** Side channel of Awash R. at Kada Bada (site 2; 10°13'53"N, 40°34'43"E; 562 m a.s.l.), type locality of *E. yardiensis* sp. nov. **B** Lake Yardi (site 1; 10°15'10"N, 40°32'9"E; 562 m a.s.l.).

Specimens were abundant in shallow shoreline habitats of the main channel (low flow velocity), deep (max. 1.5 m) stretches of side channels, stagnant water bodies of the adjacent floodplain, and the shoreline of lakes (Fig. 11). A preference for structured habitats with aquatic plants, woody debris, dense river bank vegetation and fine substrate (sand and finer fractions) was noted. A pronounced difference between wet and dry season is characteristic for the area. The water was usually turbid (suspended solids). Water temperature ranged from 26.1 °C to 31.9 °C.

Gut contents examined from type locality (*N* = 15) contained unidentifiable fine organic detritus and remains of planktonic crustaceans (exuviae of Phyllopoda (Cladocera), Copepoda, Rotatoria) but also nematodes, remains of terrestrial arthropods (beetles), diatoms, larger plant material (e.g., leaf parts), seeds, and wood debris, and some non-organic material (sand grains).

The lowland fish fauna of the Awash River is dominated by cyprinids (*E. yardiensis* sp. nov., *Garra makiensis* (Boulenger, 1903) (in Boulenger 1903b), *G. dembeensis* (Rüppell, 1835), *Labeobarbus intermedius* (Rüppell, 1835) and introduced *Cyprinus carpio* Linnaeus, 1758 but also includes *Micropanchax antinorii* (Vinciguerra, 1883), *Oreochromis niloticus* (Linnaeus, 1758), Coptodon cf. zillii and *Clarias gariepinus* (Burchell, 1822) (Golubtsov et al. 2002; Englmaier 2018).

##### Etymology.

The species name *yardiensis* refers to Lake Yardi, where the new species is abundant.

##### Comparative remarks.

Our data confirm the assumption that *E. yardiensis* sp. nov. belongs to the group of *E. paludinosus*-like smiliogastrin barbs. Based on data from Greenwood (1962) and Golubtsov and Berendzen (2005) (the latter for *E. kerstenii* from Lake Chamo-Abaya basin), the new species is different from the *E. kerstenii* complex by the absence of an orange or yellow blotch on the operculum (vs. presence) and 32–35 total lateral-series scales (vs. 23–27 in the lateral line that equals to lateral series in case lateral line is complete).

*Enteromius yardiensis* sp. nov. clearly differs from all examined species (Tables 2–5) and still unidentified forms (or undescribed species) in the group of *E. paludinosus*-like fishes by the absence of the anterior barbel, the absence of the medial branch of the supraorbital cephalic canal and few, 1–3, commonly two, scale rows between lateral line and anus.

**Table 5.** Number of serrae on last unbranched dorsal-fin ray in *Enteromius yardiensis* sp. nov., *E. akakianus*, *E. pleurogramma*, *Enteromius* sp. CMER, *E. paludinosus*, and *E. longicauda*. Values with * indicating counts found in holotypes and lectotypes. CMER referring to Central Main Ethiopian Rift as defined in text. Values are minimum - maximum and mean in parentheses. Information per specimen as in Table 1.

	< 25 mm SL	25–30 mm SL	30–35 mm SL	35–40 mm SL	40–45 mm SL	45–50 mm SL	50–55 mm SL	55–60 mm SL	60–65 mm SL	65–70 mm SL	> 70 mm SL
*E. yardiensis* n = 39	8–11 (10)	9–15 (13)	15–19 (17)	17–21 (20)	20*–24 (22)	20	27				
*E. akakianus* n = 3										24–26* (25)	35
*E. pleurogramma* n = 3		10	12	13							
*Enteromius* sp. CMER n = 32			10–12 (11)	12–13 (13)	14–18 (16)	14–18 (16)	15–20 (17)	15–20 (17)	15–19 (17)		22
*E. paludinosus* n = 17				11	15–19 (18)	19–20 (20)	20–23 (21)			20–23 (21)	27*
*E. longicauda* n = 3									20	20–23* (22)	

**Comparison of *E. yardiensis* sp. nov. with Ethiopian congeners.** Besides the characters mentioned above, *E. yardiensis* sp. nov. is readily distinguished from the *E. pleurogramma* syntypes (Lake Tana basin, Upper Blue Nile) by a set of characters: commonly seven branched pelvic-fin rays (vs. eight); 33–34 total vertebrae (vs. 35); 17–18 abdominal vertebrae (vs. 19–20); 6–9, commonly seven, vertebrae between first pterygiophores of the dorsal and anal fins (vs. 8–9, commonly nine); 32–35, commonly 33, total lateral-series scales (vs. 34–36); and 1–3, commonly two, scale rows between the lateral line and the pelvic-fin origin (vs. four) (Table 4).

Literature data confirm the distinctiveness of the new species and *E. pleurogramma* from Lake Tana which is characterised by 7–9, commonly eight, branched pelvic-fin rays; 34–36, commonly 35, total vertebrae; 32–37, commonly 35, total lateral-line scales; and 4–6 scale rows between the lateral line and the pelvic-fin origin (Dejen et al. 2002; Golubtsov and Berendzen 2005).

**Figure 12.**
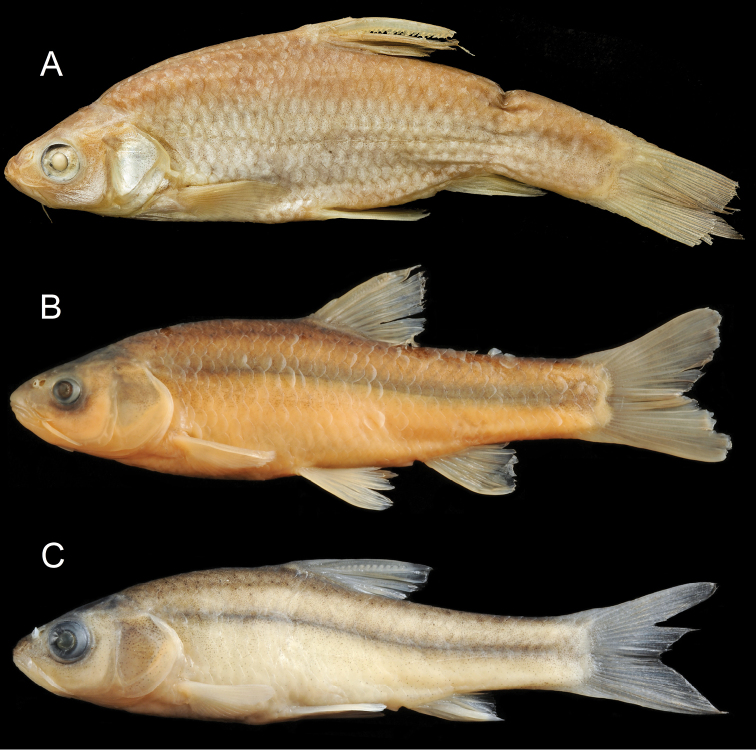
General appearance of **A***Enteromius akakianus* (same specimen as in Fig. 10B) The Trustees of the Natural History Museum, London **B***Enteromius* sp. CMER, NMW 99239, Labo R., Meki R. drainage (site 6), 70.4 mm SL **C***Enteromius* sp. CMER, NMW 99238, Lake Ziway (site 7), 45.4 mm SL.

*Enteromius yardiensis* sp. nov. can be further distinguished from *E. akakianus* (including the holotype of the latter species, Fig. 12A) by 12–14, commonly 13, branched pectoral-fin rays (vs. 16); 33–34 total vertebrae (vs. 35–37, Fig. 10B); 17–18 abdominal vertebrae (vs. 20–21); 6–9, commonly seven, vertebrae between first pterygiophores of the dorsal and anal fins (vs. 10–11); 32–35, commonly 33, total lateral-series scales (36–37); and posterior barbel coded length 1–2 (vs. 3–4) (Table 4).

*Enteromius akakianus* (Akaki River, Upper Awash drainage) is similar to the CMER combined sample (Fig. 12B, C) by most characters (Tables 3–5).

*Enteromius yardiensis* sp. nov. differs from the CMER *Enteromius* by 12–14, commonly 13, branched pectoral-fin rays (vs. 12–16, commonly 15); 33–34 total vertebrae (vs. 34–37); 17–18 abdominal vertebrae (vs. 19–20, commonly 20); 6–9, commonly seven, vertebrae between first pterygiophores of the dorsal and anal fins (vs. 8–12, commonly 10); 32–35, commonly 33, total lateral-series scales (vs. 34–37, commonly 35); posterior barbel coded length 1–2 (vs. 2–4, commonly 3) (Table 4). *Enteromius yardiensis sp. nov.* is further distinguished from the CMER samples by a wider than deep io5 (vs. deeper than wide, Fig. 9B).

We did not examine specimens from the Didessa River (tributary of the Blue Nile), the White Nile and the Omo River and refer to published data (Golubtsov and Berendzen 2005) for a comparison. These populations are identified as *E. paludinosus* (Golubtsov and Berendzen 2005) or as a member of the *E. pleurogramma* complex (Mina et al. 2017). *Enteromius yardiensis* sp. nov. can be readily distinguished by the absence of the anterior barbel (vs. always present); 12–14 branched pectoral-fin rays (vs. 13–17); 33–34, commonly 33, total vertebrae (vs. 33–36, commonly 34–35); predorsal length 53–58 % SL (vs. 46–55 % SL). Data on cytb located Didessa *Enteromius* close to *E. pleurogramma* but as a still distant (p-Euclidean distance 6.6–6.8 %) and well supported clade (Fig. 3, Suppl. material 1, Table S4).

**Figure 13.**
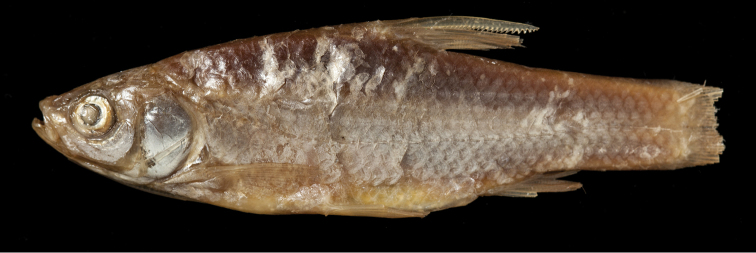
General appearance of *Enteromius paludinosus* (same specimen as Fig. 10C) The Trustees of the Natural History Museum, London.

**Comparison of *E. yardiensis* sp. nov. with East African congeners outside Ethiopia.** All morphological analyses of the type series of *E. paludinosus* and *E. longicauda* (both are from the Lower Zambezi) revealed their closest morphological affinities. This brings additional support to Greenwood’s (1962) and Seegers’ (1996) synonymisation of the two species, with priority to the name *paludinosus*. *Enteromius yardiensis* sp. nov. differs clearly from *E. paludinosus* samples (which combine the type specimens of *E. paludinosus* and *E. longicauda*) (Fig. 13) by the absence of the anterior barbel (vs. presence) and a set of counts (Table 4), including fewer, 1–3, mean 2.2, transversal scale rows between the lateral line and the pelvic-fin base (vs. 3–4) and between the lateral line and the anus, 1–3, mean 2.1 (vs. 4–5); fewer total vertebrae, 33–34, mean 33.2 (vs. 34–36, mean 34.3); and a higher number of predorsal abdominal vertebrae, 10–12, mean 10.9 (vs. 9–10, mean 10.0). The ranges of number of lateral-series scales largely overlap (32–35, mean 33.3 vs. 33–37, mean 34.5), but the highest count, 36 and 37, recorded in *E. paludinosus* were not found in the new species. A character distinguishing the two species is also the structure of the last unbranched dorsal-fin ray: in *E. yardiensis* sp. nov., the lower (non-serrated) part of the ray is short (less than one-third of the entire unsegmented part of the ray) (Fig. 10A) vs. a markedly longer (much longer than one-third) lower non-serrated part of the ray in *E. paludinosus* (Fig. 10C). Respectively, in *E. yardiensis* sp. nov., the upper serrated part is commonly longer than 75 % of the entire unsegmented part of the ray (vs. 59 % and less in *E. paludinosus*).

*Enteromius amphigramma* (Nairobi River, Kenya [Nairobi River, Kilimanjaro]), *E. loveridgii* (Amala River, Kenya), *E. macropristis* (Lake Victoria), *E. macropristis meruensis* (Mount Meru, Tanzania) and *E. vinciguerraii* (Wembere River, Tanzania) are currently synonymised with *E. paludinosus.* The type series of these nominal species examined in the present study showed that they are different from *E. yardiensis* sp. nov., first of all, by the presence of the anterior barbel, which is well-developed in all species including small-sized *E. vinciguerraii*.

*Enteromius yardiensis* sp. nov. shares with *E. macropristis meruensis* and *E. vinciguerraii* such characters as a high number of predorsal abdominal vertebrae (10–12) and a lower number of vertebrae between the first pterygiophores of the dorsal and anal fins (6–9). However, the new species is well distinguished from the two by fewer vertebrae: 33–34 total and 17–18 abdominal (vs. 35–36 and 19, respectively) and the absence of a small distinct dark spot at the end of the caudal peduncle (vs. presence).

Within the group of small-sized African smiliogastrin barbs with a thickened and serrated last unbranched dorsal-fin ray outside Ethiopia, a very short or vestigial anterior barbel was reported in *E. apleurogramma* (Boulenger, 1911) from Lake Victoria, *E. amboseli* (Banister, 1980) from the Middle Athi River in Kenya (Boulenger 1911, Banister 1980, Schmidt et al. 2018), and specimens identified as *E. paludinosus* from Satansplatz in South Africa (nowadays Namibia, Orange River drainage, Atlantic basin) (Greenwood 1962: 162). Neither *E. apleurogramma* nor *E. amboseli* has been reported from Ethiopian drainage systems. *Enteromius yardiensis* sp. nov. is readily distinguished from both species by a complete lateral line (vs. incomplete) and a higher number of lateral-series scales (32–35 vs. 20–25).

## Discussion

Combined morphological and mitochondrial data obtained in this study clearly show a distinctiveness of the Lower Awash *E. yardiensis* sp. nov. from *Enteromius* species distributed in the CMER region. This latter form, as shown above, was supported as a distinct unit on the species level.

The two most distinguishing characters, the absence of the anterior barbel and the absence of the medial branch of the supraorbital sensory canal, are both apparently specialisations (derived states) representing reductions of structures commonly present in the studied group of species.

The conclusion that the absence of the anterior barbel in all examined specimens of different size (8.0–52.8 mm) is a secondary reduction in the new species, is supported by the presence of a respective foramen in the maxillary for the maxillary branch of the trigeminal nerve innervating the anterior barbel (in species with the anterior barbel present). The presence of the anterior (rostral) barbel and the maxillary foramen for the nerve are assumed apomorphies of the subfamily Cyprininae (now at the family level) of the family Cyprinidae (Howes 1981). The secondary reduction of the anterior barbel might be related to the small size of *E. yardiensis* sp. nov. Greenwood (1962) recorded the reduction of the anterior barbel in *E. paludinosus* from Satansplatz. However, small-sized *E. vinciguerraii* and, apparently, small-sized *E. paludinosus*-like fishes (maximum SL does not exceed 40 mm) possess a well-developed anterior barbel. In *E. vinciguerraii*, the anterior barbel is present in smallest examined specimens (SL less than 30 mm). Barnard (1943: 172) analysed a series of small *E. paludinosus*-like fishes from the Fish River at Aiais, South West Africa, Orange River drainage (nowadays Namibia) and found that the anterior barbel was already developed in fishes 29–30 mm long (probably TL).

The medial branch, even a very short segment, of the supraorbital canal was not found in the examined material of the new species. In all other examined species, it was present though variably long – the longest state is the terminal pore of the branch located at the frontal parietal border and the shortest is the branch reduced to a tiny canaliculum. Among the examined set of species, the cephalic sensory canal pattern (disjunctions between the canals and the lack of particular canal segments, for example, on the operculum) is very diverse. It is much more variable than described by Skelton (1980), for South African redfin barbs, who distinguished two stable patterns: type A with 1) the preopercular-mandibular and infraorbital canal connected with the operculum and 2) the medial branch of the supraorbital canal present (serrated-rayed redfins); and type B with 1) the preopercular-mandibular and infraorbital canal disconnected and 2) the medial branch of the supraorbital canal absent (flexible-rayed redfins) (Skelton 1980: fig. 3.48). The cephalic canal pattern found in *E. yardiensis* sp. nov. belongs to Type B though the species is characterised by a thickened and serrated ray in the dorsal fin.

**Figure 14.**
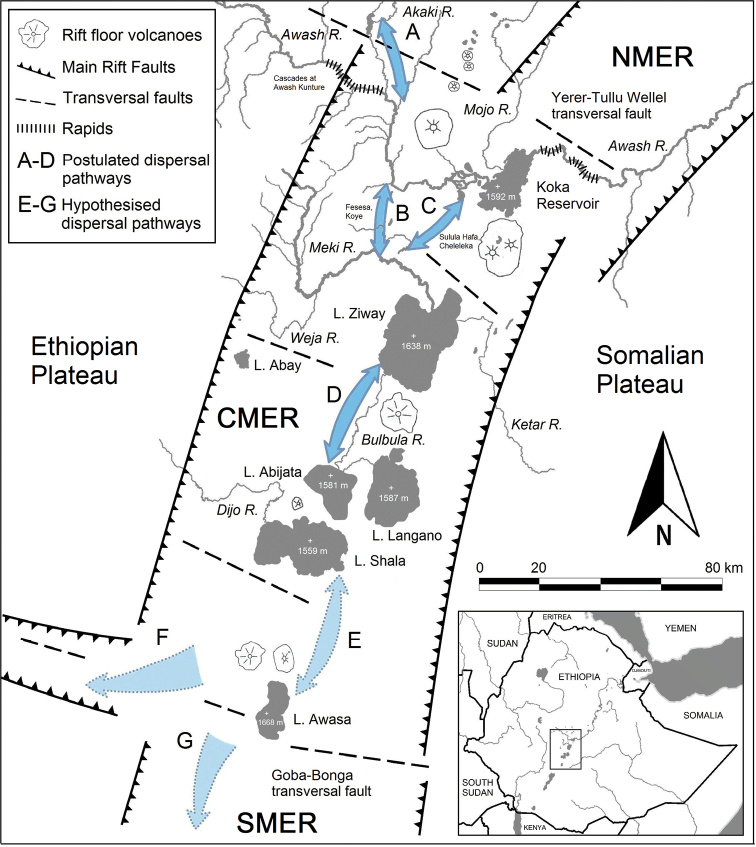
Dispersal pathways (**A–G**) and tectonic setting in Central Main Ethiopian Rift. (**A**) pathway into Akaki R. (**B**) Fesesa and Koye paleovalleys (**C**) Sulula Hafa, and Cheleleka palaeovalleys (**D**) interconnection between CMER lakes (**E**) pathway into L. Awasa (**F**) connection to Omo drainage (**G**) connection to Southern Main Ethiopian Rift. Based on Bonini et al. (2005); Sagri et al. (2008: figs 6, 12); Maslin et al. (2014); and Benvenuti and Carnicelli (2015: fig. 17.13).

As *E. yardiensis* sp. nov. is not conspecific with *E. akakianus*, the important issue was to identify the CMER specimens. No clear morphological difference was found between the holotype and topotypical specimens of *E. akakianus* and the CMER *Enteromius*, so, we preliminary identify the latter as *E. akakianus*. However, there is no genetic data available at present to check this hypothesis. We did not manage to collect *Enteromius* in the Upper Awash River and its tributaries downstream to the Koka Reservoir (Fig. 1, unnumbered localities: Chilimo Forest, Gare Arera, Awash Belo, Awash Kunture, Sulula, Lafessa) (see also Englmaier 2018: fig. 4a). Furthermore, small-sized smiliogastrin barbs with a serrated last unbranched dorsal-fin ray were not found in the entire Upper Awash by other authors either (Getahun and Stiassny 1998, Golubtsov et al. 2002). An upstream migration of *Enteromius* to the source region of the Awash River is supposedly blocked by the chain of cascades at Awash Kunture (Fig. 14) that was established at least with the onset of the rifting process at approximately 6–5 Ma (Bonini et al. 2005). It was shown that the upstream dispersal of the fish assemblages in the Awash River is considerably influenced by these cascades (Englmaier 2018).

So far, the only known locality in the Upper Awash is the type locality of *E. akakianus*, the Akaki River. It is a tributary to the Awash River downstream of the Awash Kunture rapids. This might explain why *Enteromius* could penetrate into the Akaki River from the CMER (Fig. 14). The Akaki River was sampled by GE and GT in March 2019, but no *Enteromius* were found. The river is heavily polluted by domestic and industrial sewage from Addis Ababa (see also Golubtsov et al. 2002).

In geological scales, the recent Awash River course is relatively young (Sagri et al. 2008) and the ancient Upper Awash system (upstream of the Koka Reservoir) was situated within the northern part of the CMER (Sagri et al. 2008; Benvenuti and Carnicelli 2015). Paleo-hydrological data indicate that the Upper paleo-Awash emptied into the lake basin of the CMER at least until the beginning of the Holocene (Sagri et al. 2008; Benvenuti and Carnicelli 2015). Connections between the recent Upper Awash drainage and the lakes in the CMER via rivers (now dry valleys Fesesa, Koye, Sulula Hafa and Cheleleka, Fig. 14) are well documented. Sediment records date the lacustrine (Megalake and Makrolake) phases in the Pleistocene at 100,000–22,000 years BP and in the Holocene at 10,000–5,000 years BP (Laury and Albritton 1975; Street 1979; Le Turdu et al. 1999; Sagri et al. 2008; Benvenuti and Carnicelli 2015).

These data allow us to hypothesise that the lower part of the Upper paleo-Awash system including the Akaki River was in contact with the CMER at least until the beginning of the Holocene providing pathways for fish dispersal (Fig. 14). Fish taxa shared by the Upper Awash and the CMER include *Garra makienis*, *G. quadrimaculata*/*aethipica* complex, *G. dembeensis*, *Labeobarbus intermedius*, *Oreochromis niloticus*, *Clarias gariepinus*, *Micropanchax antinorii* and an *E. paludinosus*-like smiliogastrin barb (Golubtsov et al. 2002; Stiassny and Getahun 2007; Vijverberg et al. 2012; Englmaier 2018). However, *L. ethiopicus* (Zolezzi, 1939) endemic to the Lake Ziway basin and *L. beso* (Rüppell, 1835) distributed in the Awash downstream to Nur Sada and in the Blue Nile may indicate a more complex scenario of vicariant and dispersal events.

Another important aspect is the pattern of geographic distribution of *E. yardiensis* sp. nov. and substantial level of morphological and genetic divergence from *E. akakianus* in the concept accepted above with little evidence of any gene flow.

In general, distribution of *Enteromius* in endorheic basins of central Ethiopia is congruent with its geological and zoogeographical delineations. The MER is a geologically heterogeneous system that was traditionally differentiated into three main segments: (1) the Southern (SMER), (2) the Central (CMER), and (3) the Northern MER (NMER) (Bonini et al. 2005 and references therein). The NMER extends from the Afar Depression south-west to the Yerer-Tullu Wellel major transversal fault following the middle course of the Awash River valley. The CMER encompasses most of the Lakes Region from the Koka Reservoir south to the Lake Awasa area separated from the SMER by the Goba-Bonga major transversal fault (Fig. 14). The SMER is not clearly separated from the Kenyan Rifts extending in the south into a system of basins and ranges referred to as the broadly rifted zone of Southern Ethiopia connected with both the Kenyan Rift and the Albertine (Western Branch) Rift (Bonini et al. 2005; Corti 2009; Mairal et al. 2017).

*Enteromius paludinosus*-like fishes are absent from the SMER. This area is inhabited by an *E. kerstenii*-like species (Lakes Chamo-Abaya) and at least two species without a serrated last unbranched dorsal-fin ray (Lakes Chamo-Abaya, Lake Chew Bahir, Lake Turkana) (Seegers et al. 2003; Golubtsov et al. 2002; Golubtsov and Habteselassie 2010). Zoogeographically, the SMER belongs to the Nilo-Sudan Province of Roberts (1975) and Paugy (2010) which is characterised by a higher number of species including typical nilotic elements when compared to the fish fauna of the CMER and NMER. These two latter areas are part of the Ethiopian Rift Valley Province (Paugy 2010: fig. 5).

CMER and NMER both have their individual, geographically isolated, species of *E. paludinosus*-like smiliogastrin barbs. *Enteromius yardiensis* sp. nov. was found only in the wetland area at Gewanae - site 1 (Lake Yardi) and site 2 (Kada Bada) and further downstream (Fig. 1) (approximately 400 km between the CMER and Gewanae). It was absent from five sampled localities in the CMER and the NMER (Wonji, Korkada, Nur Sada, Yimre, and Worer) downstream to Gewanae (Englmaier 2018: fig. 4b).

An isolation of the Lower paleo-Awash from the CMER occurred between the latest Pleistocene (100,000 years BP) and early Holocene (5,000 years BP), as indicated by paleo-hydrological data (Sagri et al. 2008; Benvenuti and Carnicelli 2015). The recent course of the Awash was established in the mid-Holocene and followed an opening of the Afar Depression accompanied by volcanic activities (Sagri et al. 2008). The Upper paleo-Awash system was subsequently disconnected from the CMER lakes (Sagri et al. 2008) and followed the rift to the north-east (connection to the Lower paleo-Awash drainage). River networks in earlier geological stages of the CMER are still unknown, but the course of the paleo-Awash drainage must have been subject to frequent changes (Gallotti et al. 2010). Tectonic and volcanic activities, geomorphological changes (erosion, downcutting) and a changing paleoclimate (wet and dry periods) are well documented for the MER until the Miocene (Kalb et al. 1982; Kalb 1995; Benvenuti et al. 2002; Abebe et al. 2007; Gallotti et al. 2010; Abbate et al. 2015; Benvenuti and Carnicelli 2015). This has not only affected distribution patterns of terrestrial animals (e.g., Bibi et al. 2017) and vegetation cover (e.g., Bonnefille et al. 2004; WoldeGabriel et al. 2009; Bibi et al. 2017) but presumably also the evolution of the river networks.

The presence of fish in the Lower paleo-Awash is known from excavations in the area of Gewanae which date back to the Miocene (Murray and Stewart 1999; WoldeGabriel et al. 2009; Stewart and Murray 2017). In late Miocene deposits, the earliest fossil evidence of a cyprinid with a serrated dorsal-fin ray in the paleo-Awash corridor was discovered (Stewart and Murray 2017). This is well in accordance with the formation of the MER at approximately 5 Ma BP (Bonini et al. 2005). However, it is uncertain if this fossil record represents an ancestor of small-sized African smiliogastrin barbs (Stewart and Murray 2017).

To summarise, the results of the present study provide solid support for some conclusions. First, Ethiopian *Enteromius* species with a serrated dorsal-fin ray are distant from true *E. paludinosus* (with *E. longicauda* as a synonym) and the so-called *E. paludinosus* complex involves several distinct species in accordance with molecular data of Schmidt et al. (2017). Second, two distinct species occur in the Main Ethiopian Rift area – a new species, *E. yardiensis* sp. nov., endemic to the Afar Depression in the north-eastern part of the NMER, and *E. akakianus*, endemic to the CMER lake region and the lower reaches of the Upper Awash River. An integrated approach combining genetic markers and a variety of morphological methods based on a wide set of characters, including osteology and sensory canals, proved to be very productive for taxonomy in this group of fishes.

## Supplementary Material

XML Treatment for Enteromius yardiensis

## Supplementary materials

Supplementary material 1 Supplementary tables S1–S10 Data type: species data Explanation note: **Table S1.** Voucher specimens included in present study, with GenBank accession numbers and localities. **Table S2.** List of character states (morphometric characters, coded qualitative characters, and meristic characters). **Table S3.** Primary data (morphometric and meristic) for all examined specimens. Values highlighted in blue mark cases where means used to substitute missing data; characters in red not used for statistical analyses (because of incomplete data); blank spaces indicating missing data. **Table S4.** Pairwise genetic distances (p-distances) for CO1 and cytb. **A** comparison of 611 bp of CO1 between Ethiopian populations and *Enteromius paludinosus* from Lower Zambezi River (LT629216, LT629217, locality closest to type locality of *E. paludinosus*) **B** comparison of 520 bp of partial cytb between Ethiopian populations; (minimum-maximum and mean in parentheses). For localities see Fig. 1 and Table 1. **Table S5.** MDS: Distances in final configuration, for six samples based on means for 40 morphometric, 15 meristic and two coded qualitative characters (as in Tables 2–5). **Table S6.** PCA: Factor coordinates only containing two first factors (82.6 % of variance) for 40 morphometric, 15 meristic and two coded qualitative characters (as in Tables 2–5). Most contributing characters highlighted. **Table S7.** DFA statistics (*forward stepwise*, minimal tolerance). **Table S8.** DFA: Classification matrix. **Table S9.** DFA: Squared Mahalanobis distances (distances between group centroids). **Table S10.** DFA: Standardised coefficients. Most contributing characters highlighted. https://binary.pensoft.net/file/370928This dataset is made available under the Open Database License (http://opendatacommons.org/licenses/odbl/1.0/). The Open Database License (ODbL) is a license agreement intended to allow users to freely share, modify, and use this Dataset while maintaining this same freedom for others, provided that the original source and author(s) are credited.Gernot K. Englmaier, Genanaw Tesfaye, Nina G. Bogutskaya

Supplementary material 2 Figure S1. Bayesian Inferences (BI) analysis, 520 bp of partial cytb (same data set as in Fig. 3) Data type: phylogenetic data Explanation note: Posterior probabilities from BI analysis and bootstrap (bs) values for ML (1000 bootstrap replications) above and below slash. Values below 0.70/50 considered as collapsed. Colours corresponding to those in Fig. 1. https://binary.pensoft.net/file/370929This dataset is made available under the Open Database License (http://opendatacommons.org/licenses/odbl/1.0/). The Open Database License (ODbL) is a license agreement intended to allow users to freely share, modify, and use this Dataset while maintaining this same freedom for others, provided that the original source and author(s) are credited.Gernot K. Englmaier, Genanaw Tesfaye, Nina G. Bogutskaya
