# Supplementary figures and images for: A new species of Enteromius (Actinopterygii, Cyprinidae, Smiliogastrinae) from the Awash River, Ethiopia, and the re-establishment of E. akakianus
Source: Zookeys. 2020 Jan 13;902:107–50. doi: 10.3897/zookeys.902.39606 (PMC6978609; doi:10.3897/zookeys.902.39606)

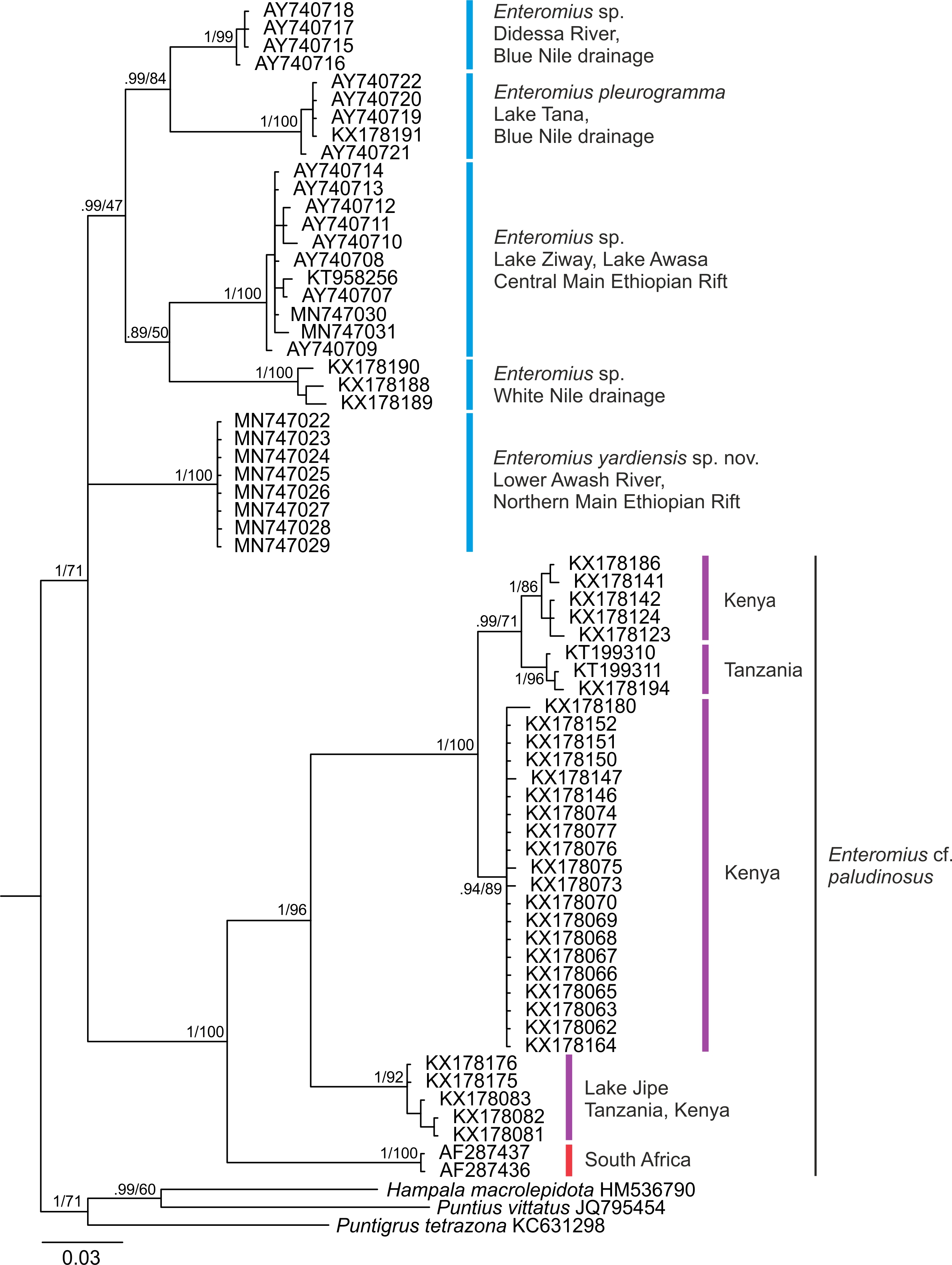

Supplement: Supplementary material 2 [file zookeys-902-107-s002.jpg]
